# Characterization of a Novel *Galactia lindenii* Lectin and Its Effects on Lepidopteran Midgut Cells

**DOI:** 10.3390/ijms262110359

**Published:** 2025-10-24

**Authors:** Zulma Casas-Corredor, Edgar Reyes-Montaño, Nohora Vega-Castro, Mónica Quintero, Deisy Hidalgo-Roa, José Luis Fernández-Alonso

**Affiliations:** 1 Grupo de Investigación en Proteínas, Departamento de Química, Facultad de Ciencias, Universidad Nacional de Colombia, Bogotá 111321, Colombia; navegac@unal.edu.co (N.V.-C.); mquintero@unal.edu.co (M.Q.); djhidalgor@unal.edu.co (D.H.-R.); 2 Real Jardín Botánico CSIC, Biodiversity and Conservation Department, Plaza de Murillo 2, 28014 Madrid, Spain; jlfernandeza@rjb.csic.es

**Keywords:** Fabaceae, lectin, *Galactia lindenii*, glycans, insecticides, *Spodoptera frugiperda*

## Abstract

Lectins are carbohydrate-binding proteins involved in plant defense and have been widely explored for their insecticidal properties. While most lectins studied to date target pests from the orders Homoptera and Hemiptera, few are effective against Lepidoptera. This group includes highly destructive agricultural pests such as *Spodoptera frugiperda*. In this study, we report the purification and characterization of a novel Glc/Man-specific lectin, GLL-I, from the seeds of *Galactia lindenii*, an endemic Colombian legume. GLL-I was purified using ion exchange and affinity chromatography, and SDS-PAGE, isoelectric focusing, and mass spectrometry confirmed its identity. Structural analysis revealed a canonical legume lectin fold with high sequence similarity to ConA lectin. Functional assays demonstrated that GLL-I exhibits dual effects on CF203 insect cells derived from *S. frugiperda*, promoting proliferation at a low concentration (0.03 μM) and inducing cytotoxicity at a higher concentration (10 μM). Histochemical analyses confirmed the binding of biotinylated GLL-I to the midgut of *S. frugiperda* larvae. These findings suggest that GLL-I differs from previously characterized lectins in its origin and target specificity. It highlights its potential as a novel bioinsecticidal agent for controlling lepidopteran pests affecting key crops such as maize.

## 1. Introduction

Lectins are proteins with domains that reversibly recognize and bind carbohydrates. They are found in various organisms, including animals, plants, bacteria, fungi, algae, and viruses, among others [1,2]. Lectins participate in multiple cellular processes mediated by receptor–ligand, macromolecule–cell, cell–cell, and cell–extracellular matrix interactions. These processes facilitate the intracellular formation of multimeric complexes, adhesion, and intercellular recognition. Due to these functions, lectins have been proposed as potential therapeutic agents and are of significant interest for various biotechnological applications [1,3].

Some plant-derived lectins have been used as models to study protein–carbohydrate interactions. Among them, lectins from the Leguminosae family are highly relevant due to their diverse biochemical properties, biological functions, and carbohydrate-binding specificity [4]. These proteins exhibit similarities in their primary structure and physicochemical properties. They are classified in families because they recognize various carbohydrate structures according to their structural motifs [5,6]. The families of legume-type lectins (type-L), galectins, and pentraxins share a β-sandwich fold but differ in the number and assembly of their subunits. Thus, several L-type lectins and galectins can exist as monomers or dimers, while pentraxins and galectin-3 are found as pentamers. Regarding their distribution, the L-type family is found in plants and fungi, while the galectin family is present in animals, viruses, and fungi. In conclusion, these proteins share the same three-dimensional structure, but their structural motifs are distinct, and, to date, 48 families have been described [5].

Despite having a conserved primary structure across different species, L-type lectins have broad recognition specificity for various carbohydrate residues. The available sequences (RCSB_PDB, UniprotKB/Swiss-prot) show approximately 20% identical amino acids and 40% similarity. They generally comprise two or four identical subunits with a monomeric molecular weight ranging between 25 and 30 kDa, each containing binding sites for metal ions such as Ca^2+^, Mn^2+^, and Mg^2+^. L-type lectins can form dimeric, tetrameric, or oligomeric structures [7]. This group of lectins is found in species belonging to the Diocleae tribe, comprising 17 genera and approximately 200 species [8].

The plant species exhibit primitive characteristics such as a woody habit, trifoliate leaves, stipules, racemose inflorescences, and large flowers with a hypanthium [8,9]; the non-protein amino acid canavanine is present. A molecular phylogenetic analysis based on nuclear and chloroplast markers identified three clades within the Diocleae Hutch tribe: Canavalia (containing species of the genus *Canavalia* DC.), Dioclea (genera *Dioclea* Kunth, *Cymbosema* Benth, *Cleobulia* Mart. Ex Benth, *Luzonia* Elmer, and *Macropsychanthus* Harms), and Galactia (genera *Galactia* P.Browne, *Neorudolphia* Britton, *Rhodopsis* Urban, *Bionia* Mart. ex Benth, *Cratylia* Mart. ex Benth, *Lackeya* Fortunato, *Camptosema* Hook & Arn., and *Collaea* DC.) [10,11].

Lectins from the Fabaceae family (Polhill) exhibit diverse biological activities, including vasorelaxant, antiproliferative, antibacterial, and insecticidal effects against Lepidoptera, Coleoptera, and Hemiptera [12,13,14,15,16,17]. Numerous studies have demonstrated the toxicity of natural proteins, such as lectins, toward pest insects, supporting their potential use as biological insecticides in agriculture [18]. Assays using artificial diets or transgenic plants expressing lectins have consistently shown reduced insect growth and development [19,20,21,22].

Among these, Concanavalin A (ConA), a lectin that binds mannose and glucose residues, has been shown to increase mortality and delay development in species such as *Helicoverpa armigera* and *Rhopalosiphum maidis* [23,24,25]. Likewise, mannose-binding lectins from plants, fungi, and algae have demonstrated potent insecticidal activity against economically important pests [26,27].

Advances in biotechnology have enabled the development of fusion lectins that enhance insecticidal efficacy by targeting the insect gut, facilitating the passage of toxic peptides into the hemolymph [28,29,30]. These results underscore the role of lectins in host defense mechanisms against pathogens and predators. At the molecular level, lectin binding to glycoproteins or other carbohydrate-containing structures can modulate enzymatic activity, receptor function, transporter availability, and growth factor signaling [31,32,33,34]. In insects, developmental stage-dependent changes in glycosylation patterns and the mode of diet delivery critically influence the efficacy and mechanism of action of lectins [21]. Lectins offer a wide range of biological activities and can specifically target insect physiology, making them a promising tool for developing sustainable pest management strategies.

The clade *Galactia* includes the genus *Galactia*, which is found in the Paleotropics and the Americas, ranging from the United States to central Argentina, with 50 species. These nitrogen-fixing symbiotic plants grow in poor soils and under stress conditions [35]. This genus encompasses a wide diversity of habits, root and rhizome structures, leaf venation, inflorescence structure, and floral morphology. They are generally perennial herbaceous plants, mostly vines. The genus includes 38 species in South America [35,36]; of these, *Galactia lindenii* Burkart is endemic to Colombia and of particular interest in this study, as its seeds contain two lectins with different physicochemical properties and recognition patterns: GLL-I (Type I), which recognizes Man/Glc, and GLL-II (Type II), whose activity is inhibited by the monosaccharide N-acetylgalactosamine (GalNAc). Similarly, two analogous lectins have been found in Canavalia and Dioclea clades species, such as *Canavalia brasiliensis* Mart. ex Benth (ConBr), *Dioclea grandiflora* Mart. ex Benth. (DGL), *Dioclea guianensis* Benth. (DguiL), and *Canavalia ensiformis* (L.)DC. (ConA) [35,36,37,38,39,40,41].

Dioclea-type I lectins are oligomers formed by identical monomers of ~25–30 kDa, and their equilibrium for dimer and tetramer formation is pH-dependent. They are generally metalloproteins that require divalent ions (Ca^2+^ and Mn^2+^) for their biological activity [42] and have a high identity in their primary structure. In some cases, they may exhibit specific differences in amino acid sequences or alternative conformations, leading to significant variability in their biological activities [43,44]. Mannose (Man) and/or Glucose (Glc) inhibit agglutination activity; therefore, it is purified by affinity chromatography using dextran resin [7]. The molecular characteristics are similar, with differences in recognizing various mannose-type oligosaccharides. Regarding the lectins from the genus *Galactia* studied to date, the lectin from *G. lindenii* II, GLL-II, is the most well-characterized at the molecular level [45,46]. The study on GLL-II was the first to provide evidence of a second lectin present in the Diocleinae tribe, distinct from the Glc/Man-specific type, which specifically recognizes the H-type 2 human blood group determinant.

In this context, *Galactia lindenii*, a member of the Fabaceae family, stands out as a potential new source of bioactive lectins. Researching the biological activities of GLL-I could lead to the discovery of novel insecticidal compounds, thereby expanding the range of biotechnological tools available for agricultural pest control. Additionally, understanding the specific mechanisms of action for these lectins, especially insect glycosylation patterns and dietary delivery methods, will be crucial for optimizing their effectiveness and application.

## 2. Results and Discussion

### 2.1. Extraction and Purification

The protein extraction process began with 5 g of flour. Thiourea was added to the extraction buffer at a concentration of 5 mM to inhibit polyphenol oxidase activity. This step is crucial as it helps minimize the pigmentation in plant extracts caused by polyphenol oxidation [47]. By the end of the process, the protein extracts were soluble in PBS (20 mM phosphates, 150 mM NaCl) at pH 7.25, exhibiting low pigmentation and viscosity, along with agglutination activity toward human A and O erythrocytes (Table 1).

Extraction of proteins using PBS yielded 268.6 mg of soluble protein per gram of flour. In contrast, when using a buffer of 100 mM acetate, 150 mM NaCl, and 5 mM thiourea at a pH of 5.0, the soluble protein was significantly lower, yielding only 67.2 mg per gram of flour. These findings differ from previous studies that reported more than 153 mg of protein extracted per gram of flour under similar conditions, using 10 g [46]. Furthermore, this study yielded three times less amounts than reported in another study, where 300.6 mg of total protein per gram of flour was extracted with a PBS-thiourea buffer (5 mM, pH 7.2) using 5 g of flour (Table 1) [48].

The lectins of the genus *Galactia* that recognize galactosides, such as that of *G. tashiroi* Maxim., exhibited better activity at pH 8 [49]. Its congeners could also exhibit this behavior, which would explain the higher specific titer of the crude extract at pH 7.2 of *G. lindenii* compared to that obtained at pH 5.0 (6.9 vs. 81.75) [47,48].

Differences in the protein extraction process can arise from various factors. For instance, the flour may exhibit variations in its composition due to factors such as seed maturity, milling method, moisture content, and storage conditions. These variations can affect both the quantity and quality of the proteins present in the flour, potentially requiring adjustments in the extraction process. Furthermore, procedure variations occur, as experiments are conducted to improve the efficiency and quality of the extracted proteins. Additionally, protein loss can occur during the extraction and concentration stages due to insolubilization. It has been determined that lectins from the genus *Galactia* retain their activity within a pH range of 4 to 10 [46,50,51]. Therefore, it can be emphasized that pH, the quantity and quality of flour used, the volume of the buffer employed, and the extraction duration are highly relevant factors for maintaining protein activity and improving yields in the extraction process.

**Table 1:** Purification steps, corresponding fractions, and activity of GLL-I.

Purification Step	Concentration (mg/mL) ^1^	Volume ^2^ (mL)	Total Protein (mg)	Specific Titer ^3^ Erythrocytes O+	Specific Titer ^4^ Erythrocytes A+	Total Yield (mg/g) ^5^
Total extract ^6^	6.75	199	1343.2	1.18	N.D	268.6
DEAE-FR ^7^	51.6	7	361.2	1.24	2.48	72.2
FR S200 ^8^	0.478	0.3	0.143	33.47	66.94	0.029
FPLC-II ^9^	0.510	1.7	0.867	15.68	31.37	-

^1^ Protein concentration was quantified by the BCA assay described by Smith et al., 1985 [51]. ^2^ Fraction volume total. ^3,4^ Specific titer: agglutination titer/protein concentration of the tested solution. ^5^ mg soluble protein/g flour. ^6^ Pooled extracts were concentrated in PBS 1X buffer, prepared from 5 g of flour. ^7^ Retained fraction obtained on DEAE-Sephacel column. ^8^ FR S200: fraction retained by affinity chromatography and eluted with 0.2 M glucose. ^9^ FPLC-II: gel filtration step; fraction containing GLL-I. Agglutination assays were performed in microplates and validated by light microscopy using 10× and 20× objectives. “N.D” specific titer was not determined.

**Figure 1:**
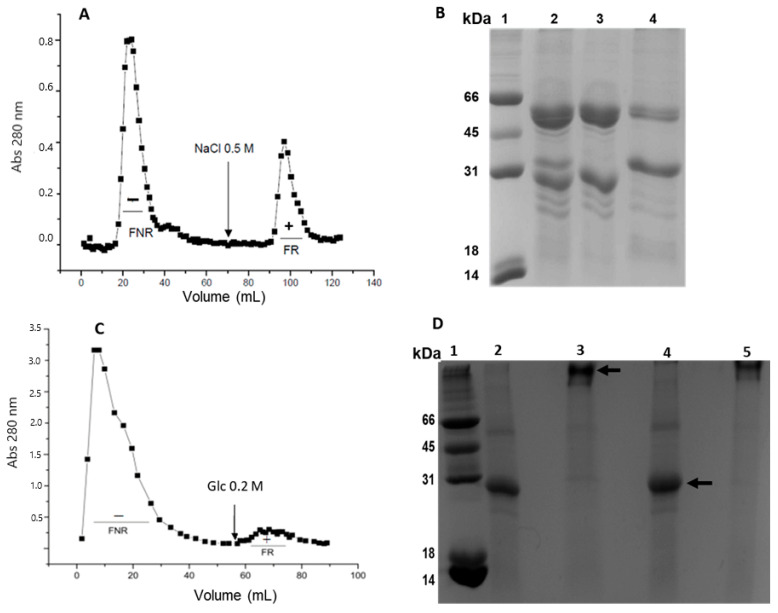
Purification of the lectin GLL-I from *Galactia lindenii*. (**A**): Chromatographic profile on *DEAE Sephadex* matrix (FNR−) unretained fraction, (FR+) retained fraction. (**B**): SDS-PAGE analysis of ion exchange chromatography (20 μg of protein per lane), staining with Coomassie Brilliant Blue R-250. **Lanes**: 1. Molecular weight marker (kDa), 2. crude extract, 3. unretained fraction ion exchange chromatography, 4. retained fraction ion exchange chromatography. (**C**): Affinity chromatography using a Sephacryl S-200 matrix. (**D**): SDS-PAGE (15 μg of protein per lane), stained with Coomassie Brilliant Blue R-250. GLL-I under different conditions. 1. Molecular weight marker (kDa). 2. + SDS +SDS, with heating (Δ). 3. +SDS, without heating. 4. +SDS + DTT, with heating (Δ). 5. +SDS + DTT, without heating. Arrow in 1D shows the 30 kDa monomer.

In the initial purification stage, ion exchange chromatography was performed using DEAE-Sephadex (Figure 1A). This technique proved advantageous for separating the two lectins in the crude extract (Figure 1B, lane 2), as they possess differentiated isoelectric points. The unretained fraction (FNR) contains GLL-II (Figure 1B, lane 3), specific for the H2-type trisaccharide (Fucα1,2Galβ1,4GlcNAc), with an isoelectric point (pI) of 8.2 [46]. In contrast, the lectin GLL-I, specific for glucose (Glc)/Mannose (Man), was found in the retained fraction (Figure 1A (FR+)) with a pI of 6.15. The retained fraction was subjected to affinity chromatography using a Sephacryl S200 support, resulting in a fraction retained and eluted with 0.2 M Glc (Figure 1C (FR+)). Agglutination assays showed specific reactivity toward A^+^ erythrocytes over O^+^ erythrocytes, accompanied by a moderate specific titer relative to the retained fraction from DEAE-Sephadex (Table 1). GLL-I was more effective at agglutinating A+ erythrocytes, requiring a minimum of 0.625 μg of lectin for visible agglutination, while O+ erythrocytes required 1.25 μg.

SDS-PAGE under reducing conditions revealed two predominant bands around 30 and 60 kDa, corresponding to the monomer and dimer, respectively (Figure 1B, lane 4 and Figure 1D, lane 4). However, heating was necessary to dissociate the tetramer, as evidenced by a high molecular weight band and the complete absence of the monomer and dimer (Figure 1D, lane 3 vs. lane 4). The interaction between the lectin subunits occurs through weak, non-covalent interactions, as no differences were observed in the presence or absence of DTT (Figure 1D, lane 5 vs. lane 3). By SDS-PAGE, GLL-II shows a 24 kDa band as the main component, corresponding to the monomer, and occasionally a 50 kDa band, which corresponds to the lectin dimer due to incomplete dissociation of the tetramer (Figure 1B, lane 3) [46].

This indicates that lectins from this genus behave similarly and require heat for complete dissociation, allowing the observation of their monomeric forms despite having different specificities. For *Dioclea grandiflora* type II lectin (DGL-II), SDS-PAGE reveals a 25.4 kDa band. In contrast, DGL-I consists of an intact subunit (α) at 27.3 kDa and two fragments (β and γ) at 14.7 and 12.1 kDa, respectively [45]. Notably, for this GLL-I, the low molecular weight bands typically observed in lectins similar to ConA were not detected, suggesting that a post-translational processing may not have occurred. In many type I lectins, three bands are commonly seen in SDS-PAGE, one corresponds to the lectin monomer (α), and the other two, β and γ, are fragments resulting from partial post-translational proteolytic cleavage.

Between 3 and 4 mg of GLL-I are in 100 g of flour. According to that, 4.28 mg was recovered after purifying the crude extract using affinity chromatography on Sephacryl S-200. The micro-Kjeldahl determined the last. Type I lectins from *Dioclea lehmannii* Diels, *D. sericea* Kunth, and *D. grandiflora* are found between 1200 and 1300 mg of pure lectin per 100 g of dry flour, while ConA from *Canavalia ensiformis* contains 2400 mg/100 g of flour. In the *Dioclea* genus, type I lectins are present in higher proportions than type II lectins. Conversely, in the *Galactia* genus, type II lectins are more abundant than type I. GLL-II content in seeds was 588 mg/100 g dry flour [46]. This indicates a difference in the expression of each lectin within the *Diocleinae* tribe. These variations may be linked to the physiological functions of the seeds since lectins play a crucial role in defense against pathogens and regulating growth during germination. Furthermore, the differences in lectin proportions may reflect evolutionary adaptations specific to the environments of each genus, suggesting a direct relationship between lectin production and seed physiology. The GLL-I detection and purification results were confirmed in an independent purification assay.

**Figure 2:**
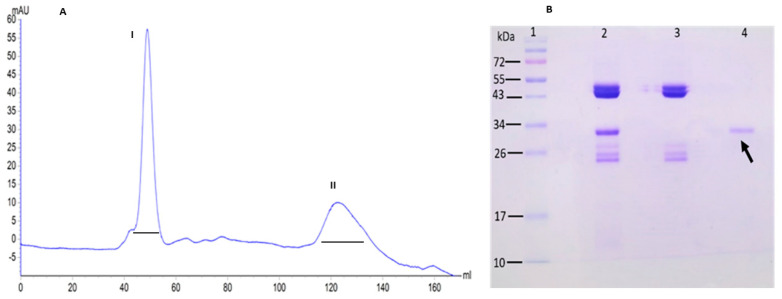
Purification of the GLL-I lectin from *G. lindenii* by size-exclusion chromatography using FPLC. (**A**). Chromatographic profile, detection at 230 nm (black lines correspond to collected fractions I and II). (**B**). SDS-PAGE analysis under denaturing and reducing conditions and heating (+Δ), stained with Coomassie Brilliant Blue G-250 (30 μg of protein per lane. Lane 1: Molecular weight markers (MW, kDa); lane 2: retained fraction (FR) from DEAE-Sephadex; lane 3: FPLC fraction I (FPLC-I); lane 4: FPLC fraction I (FPLC-II). The arrow shows purified GLL-I monomer.

The DEAE-FR underwent molecular exclusion chromatography using FPLC. The chromatographic profile revealed two main fractions. The first fraction eluted at 38 mL (Figure 2A-I), while the second emerged at 130 mL, where agglutination activity with A erythrocytes was observed (Figure 2A-II). SDS-PAGE analysis showed a distinct band at 30 kDa, corresponding to the GLL-I monomer (Figure 2B, lanes 2 and 4). The last may indicate another purification method without affinity chromatography (Table 1).

The relative mass of the GLL-I monomer, 30 kDa, is consistent with the relative mass of type I lectins similar to ConA, from the *Diocleinae* subtribe and the *Canavalia* and *Dioclea* clades, such as *Canavalia brasiliensis* (ConBr), *Canavalia grandiflora* (Jacq.) DC (ConGF), *Canavalia gladiata* Benth, and *Dioclea sclerocarpa* (Ducke) L.P. Queiroz & Snack [52,53]; these lectins associate into dimeric or tetrameric forms through non-covalent interactions. As observed by SDS-PAGE, most of these lectins display three bands: α, β, and γ. The α band corresponds to the lectin monomer. In contrast, the β and γ bands result from partial post-translational proteolytic processing by an asparaginyl endopeptidase that cleaves the Asn118-Ser119 bond during seed development. These fragments correspond to the lectin’s N- and C-terminal [54]. For *Canavalia grandiflora* (Con GF), a molecular mass of 29–30 kDa (α chain) has been reported, along with two other bands of 16–18 kDa (β chain) and 12–13 kDa (γ chain). For *Dioclea reflexa* Hook (DrfL), three bands with approximate masses of 25.5, 12.8, and 12.7 kDa, respectively, have been observed. [55]; the same occurs with the lectin from *Dioclea bicolor* Hoffmanns ex Benth. (DBL), which displays three bands, α, β, and γ, with masses of 29, 14, and 12 kDa, respectively [56]. Additionally, the Supplementary Material includes a review table of the molecular properties of these lectins (see Table S1).

For the GLL-I lectin, only the α band for the 30 kDa monomer was evident (Figure 1D, lane 4), and the same occurs with the lectin from *Cratylia mollis* Mart. ex Benth. [57]; however, for the lectin from *Cratylia floribunda* Benth., two bands are evident for the β chain (16 and 18 kDa) and one for the γ chain (12–13 kDa) [58]; they are probably in very low proportion or do not undergo post-translational modifications, and this process is not widespread in this type of lectin across all genera. In type I lectins from the genera *Dioclea*, *Canavalia*, and *Cymbosema*, the β and γ subunits are easily observed, as they are in high proportion relative to the α chain [59].

### 2.2. Carbohydrate Inhibition

The agglutination activity of GLL-I was inhibited only by the monosaccharide mannose (Man) at a concentration of 150 mM, suggesting that hydroxyl (-OH) groups at positions 2 and 4 are crucial for carbohydrate interaction and recognition, allowing discrimination between epimers. In contrast, no inhibition was observed with glucose (Glc), Galactose (Gal), Fucose (Fuc), and N-acetylgalactosamine (GalNAc) or the disaccharide lactose (Lac). Additionally, the introduction of bulky hydrophobic groups, such as p-nitrophenyl, in α or β configurations, as well as smaller groups like 1-O-methyl-β-galactopyranoside, enhances the interaction and results in the greater inhibition of agglutination (see Table S2). A relative increase in inhibitory activity was recorded compared to mannose, ranging from 2 to 4 times. The p-nitrophenyl group enhances the interaction between carbohydrates and lectins, allowing the lectin to distinguish between β and α configurations, as demonstrated by the relative inhibitory activity (16 vs. 4) (Table 2). In Fabaceae lectins, researchers have noted the presence of a region near the carbohydrate interaction site that facilitates interaction with hydrophobic groups, thereby enhancing carbohydrate recognition by the Lectin [60]. This was observed when β-methyl D-glucose, 1-O-methyl-β-galactopyranoside, β-methyl D-glucose, p-Nitrophenyl-N-acetyl-α-D-glucosamine, and p-Nitrophenyl-N-acetyl-β-D-galactosamine were used (see Table S2). Comparable behavior was reported for the DSL-I lectin (type I *Dioclea sericea*), which was also inhibited by p-nitrophenyl-β-D-mannopyranoside at the same concentration [61].

On the other hand, Dam et al. [62] conducted studies using isothermal titration calorimetry (ITC) on the type I lectin from *Cymbosema roseum* Benth. (CRL-I) revealed that its affinity for mannose-rich oligosaccharides (Ka = 24.7 × 10^4^ M^−1^) was significantly greater than for α-methyl-mannopyranoside. This increased affinity has also been observed in other type I lectins, such as Concanavalin A (ConA) and *Dioclea grandiflora* (DGL); the determined affinity constants suggested that these lectins possess an extended binding site that accommodates the tri-mannose core, a conserved binding site for these lectins [62,63]. Additionally, glycans’ structure, spatial arrangement, and complexity are crucial in determining the specificity, interaction, and affinity of type I lectins for their ligands. Inhibition assays revealed differences in carbohydrate recognition between GLL-I and GLL-II lectins. GLL-I showed higher specificity toward mannose residues, whereas GLL-II was more effectively inhibited by GalNAc, with the latter being the most potent inhibitor at 12.5 mM [46]. Affinity chromatography using Sepharose 4B-mannose did not successfully retain GLL-I. In contrast, the best results were achieved with Sephacryl S-200 as the support medium. This suggests that the carbohydrate recognition by GLL-I may depend on the accessibility or density of hydroxyl groups or that the lectin has a higher affinity for oligosaccharides than monosaccharides.

### 2.3. Determination of the Isoelectric Point and Glycosylation

**Figure 3:**
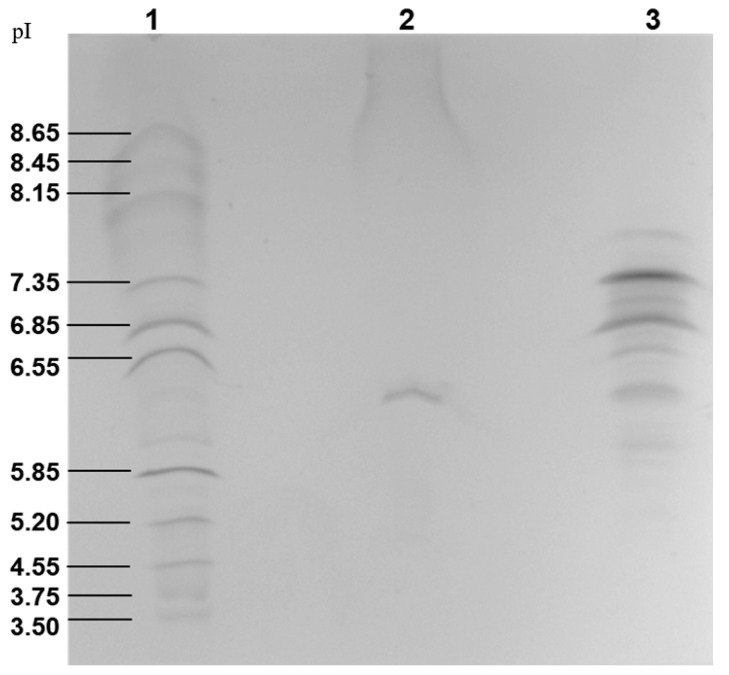
Native isoelectric focusing gel of GLL-I stained with Coomassie brilliant blue R-250. Lane 1: isoelectric point marker (pI). Lane 2: Sephacryl S-200 purified GLL-I, 20 μg. Lane 3: myoglobin 15 μg (control).

GLL-I is characterized as a glycoprotein, typical of most type I lectins studied to date. However, Concanavalin A (ConA) is a notable exception, as it lacks glycosylation [64]. The carbohydrate content in type I lectins typically ranges from 1.7% to 5%. Regarding the isoelectric point (pI) of GLL-I, a band was detected around 6.15 (Figure 3). This value is comparable to the pI reported for the *Dioclea sericea* (DSL-I) lectin, whose pI ranges from 6.6 to 6.9 [61]. In contrast, type I lectins from *Dioclea lehmanni* and *Dioclea altissima* Rock exhibited several bands in a more basic pH range, specifically between 8.0 and 8.4 and 8.6–9.0, respectively [65,66].

### 2.4. Analysis of Peptide Sequences and Determination of the Primary Structure of GLL-I in Comparison with Reported Lectins

Given the high percentage of identity among these sequences’ legume-type lectins [7], tryptic digestion was conducted. The resulting digested fragments were analyzed using nLC-MS/MS, and the results were compared through matched spectrum searches (PSM) in the Uniprot and BLAST-P databases. In our case, 298 peptides generated by PEAKS 6 de NOVO were identified; these were further analyzed with the Decrease Redundancy program from Expasy [67] to reduce the number of redundant peptides. A selected set of peptides, with an identity percentage of 90% or greater, was used for further analysis.

**Figure 4:**
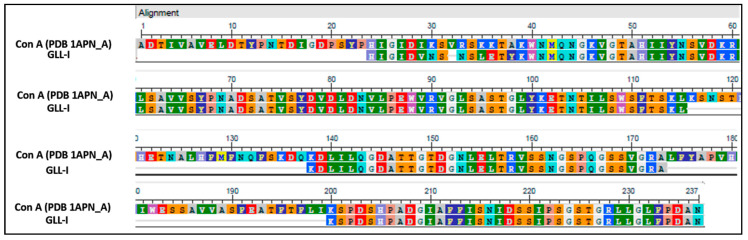
The amino acid sequence of the GLL-I lectin is aligned with Concanavalin A (ConA, PDB 1APN_A). The alignment reveals that the ten peptides exhibit a high percentage of identity with the ConA sequence. A gap is present at position 32. Polar amino acids are highlighted in bright colors, while non-polar residues are depicted in darker shades.

Subsequently, a BLASTp search was performed against reported sequences of proteins from the Canavalia and Dioclea genera, which include *Canavalia cathartica* Thouars, *C. brasiliensis*, *C. boliviana* Piper, *C. bonariensis* Lindl, *C. ensiformis*, and *Dioclea virgata* (Rich.) Amshoff. This search resulted in the selection of 10 peptides (see Table S3) (Figure 4), as approximately 67% of the amino acid residues are conserved in lectins from the Diocleinae subtribe [7].

Upon aligning the selected peptides to determine the primary structure of GLL-I, it was noted that ten of the obtained peptides were almost identical when aligned with the ConA sequence (PDB 1APN_A), with a gap present at position 32 (Figure 4).

**Figure 5:**
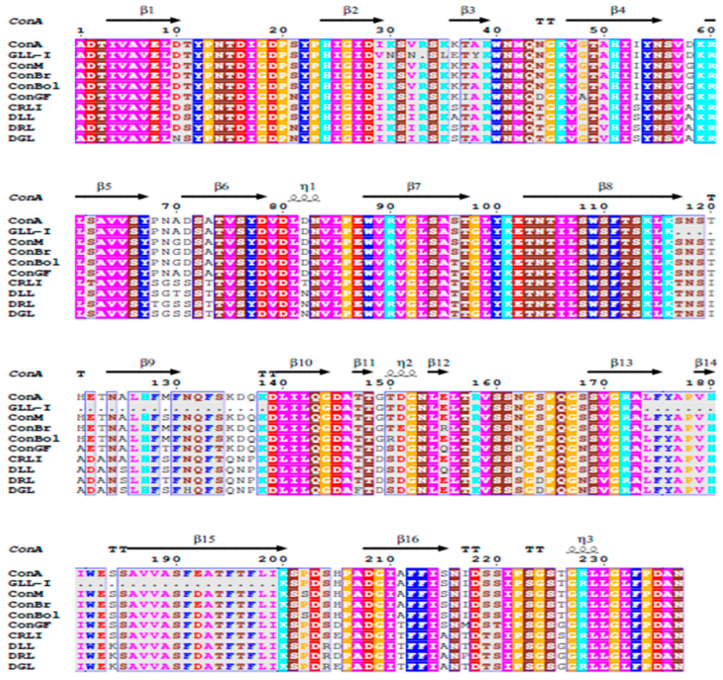
Multiple sequence alignment of the amino acid sequence of *Galactia lindenii* (GLL-I) with seed lectins from the Diocleinae subtribe: *Canavalia ensiformis* (ConA), *Galactia lindenii* type I (GLL-I), *Canavalia maritima* (ConM), *Canavalia brasiliensis* (ConBr), *Canavalia boliviana* (ConBol), *Canavalia grandiflora* (ConGF), *Cymbosema roseum* (CRL-I), *Dioclea lasiocarpa* Mart. ex Beth (DLL-I), *Dioclea rostrata* Benth. (=*Macropsychanthus bicolor* (Benth.) L.P. Queiroz & Snak) (DRL-I), *Dioclea grandiflora* (DGL-I). Colors: HKR blue-green; DE red; STNQ brown; AVLIM pink; FYW blue; PG orange; C green.

The high conservation of the N-terminal sequence in these type I lectins was assessed, and this sequence was complemented using 23 amino acid residues from ConA (Figure 5). This proposed sequence achieved 79% coverage (Figure 5), sharing 188 of the 237 amino acids reported for ConA lectin (PDB 1APN_A) (https://www.rcsb.org/structure/1val, accessed on 10 December 2023). There are still 49 amino acids to be determined, representing approximately the remaining 20% needed to complete the sequence. GLL-I contains conserved amino acids present in both the carbohydrate recognition domain (Y100, D208, and R228) and the metal-binding sites (H24) of Dioclea lectins (Figure 5). At positions 30 and 33, lysine and arginine are substituted by asparagine in both cases, representing unique substitutions not observed in any other lectin from the *Dioclea*, *Cymbosena*, and *Canavalia* genera [56].

The GLL-I sequence demonstrated a high level of similarity to lectins from the Canavalia genus, with 77% identity. In contrast, it showed a lower similarity with proteins from the Dioclea genus, with 65% identity (Figure 5). The ConV lectin from *Canavalia virosa* (Roxb.) Wight & Arn, which recognizes Man, has a 99% amino acid sequence similarity with ConA [13], while with GLL-I, it showed a lower similarity of 79%. Additionally, the sequence of the lectin from *Canavalia gladiata* exhibits similarities ranging from 81% to 98% with lectins from *C. ensiformis*, *C. brasiliensis*, *C. maritima* Thouars (=C. rosea (Sw.) DC.), *Dioclea grandiflora*, *D. guianensis*, and *Cratylia mollis* [68]. The GLL-I and GLL-II recombinant (GenBank: WAB55723.1) sequences had a 50.43% identity; the latter is specific for GalNAc. The sequencing data and the inhibition assay results support the conclusion that these are two distinct lectins.

**Figure 6:**
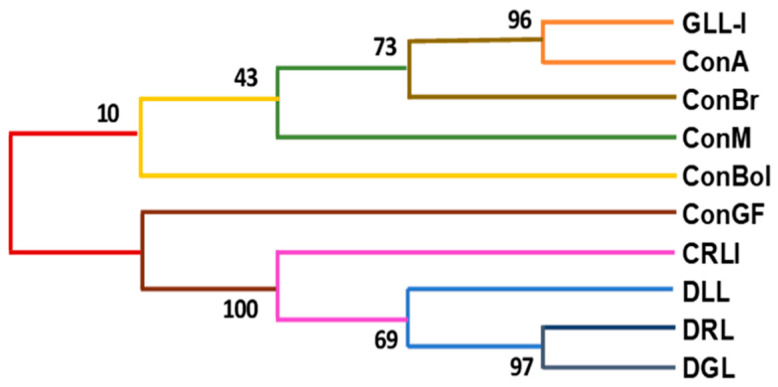
Phylogenetic tree of some legume lectins. ConA (PDB 1APN_A), ConM (PDB 2OW4_A), ConBr (sp P55915.1), ConBol (PDB 4K20_B), ConGF (PDB 4L8Q_A), CRLI: CRL-I (PDB 3A0K_G), DLL: DLL-I (PDB 5UUY_A), DRL: DRL-I (PDB 2ZBJ_A), DGL: DGL-I (PDB 1DGL_B). The lectins GLL-I and ConA clustered together with a Bootstrap value of 96, and ConBr at a Bootstrap value of 73. These are further grouped with ConM, which has a Bootstrap value of 43, and ConBol, which has a Bootstrap value of 10. The lectins DRL-I and DGL-I clustered together, with a Bootstrap value of 97 and DLL at a Bootstrap value of 69. CRLI clustered with the previous lectins, with a Bootstrap value of 100. All lectins form a single group with a Bootstrap value of 10.

The phylogenetic tree (Figure 6) shows that GLL-I and ConA are closely related, sharing a high level of evolutionary similarity, as evidenced by a Bootstrap value of 96. This high similarity may suggest similarities in their roles concerning pathogen interactions or the ecology of the plants from which they originated. The proximity of the GLL-I-ConA clade to ConBr and its greater distance from ConM and ConBol could reflect differences in the ecological niches of the species from isolated lectins or in the types of plant–insect or plant–microbe interactions each lectin facilitates. Despite these differences, all these lectins form part of a single clade, suggesting that, at a phylogenetic level, species of the genus *Canavalia* share a significant degree of conservation in the gene regions encoding these proteins.

Regarding the lectins DRL-I, DGL-I, and DLL-I, their high Bootstrap values (97 and 69, respectively) suggest a close evolutionary relationship between Dioclea species. This supports the idea that the biological functions of these lectins may also be related, potentially in defense mechanisms or symbiotic interactions. The clustering of CRL-I with these lectins, with a Bootstrap value of 100, strengthens the robustness of this relationship.

In general, the formation of a single cluster for all lectins with a Bootstrap value of 10 indicates that, although there is diversity within this group of proteins, they all share common structural or functional characteristics. This analysis suggests that, at the species level, these lectins may perform similar functions in plant physiology and their ecological interactions, such as defense mechanisms against herbivores or pathogens, reinforcing their importance in the evolution of these plant species.

### 2.5. Prediction of the 3D Structure of Lectin GLL-I

**Figure 7:**
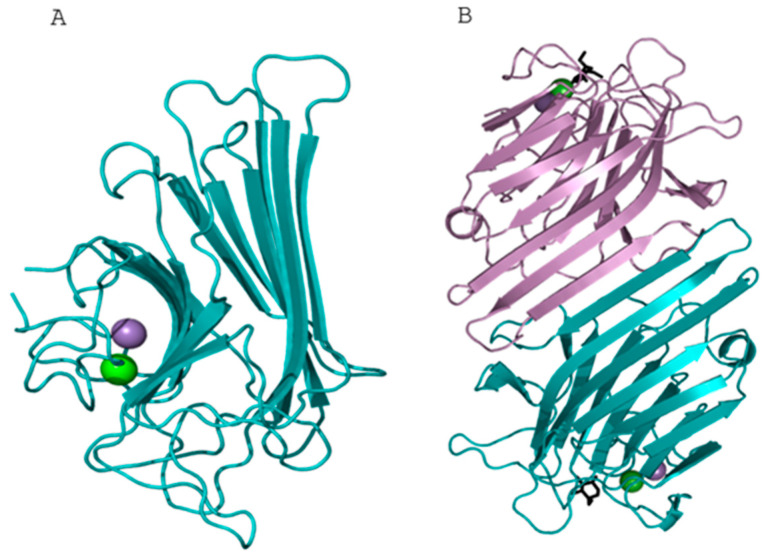
Predicted three-dimensional structure of the GLL-I lectin from *Galactia lindenii.* (**A**) The monomeric structure consists of two β-sheets forming a β-sandwich fold. (**B**) Dimeric representation showing twelve β-strands (six from each monomer) arranged antiparallelly. The monomers are colored marine blue and purple, respectively. Spheres indicate the Ca^2+^ (green) or Mn^2+^ (purple) ions bound within each monomer. Some lectins can have calcium, but others can have manganese interacting in this site.

The tertiary structure of GLL-I was modeled using Concanavalin A (ConA) from *Canavalia ensiformis* as a template (PDB:1BXH). The model demonstrated good stereochemistry, with a Ramachandran plot indicating well-defined geometry for 99% of the protein residues. The resulting three-dimensional structure exhibits a leguminous fold, typical of this family of lectins, including galectins, and mammalian pentraxins [69,70,71]. The monomer is characterized by two superimposed beta sheets, forming a β-sandwich fold [72] (Figure 7A). Generally, the number of strands that make up the β-sheets can vary among lectins [69,70,73].

In leguminous-type lectins, the β-sandwich structure consists of a curved front β-sheet composed of 5–7 β-strands and a flat rear β-sheet of 4–6 β-strands, connected on one side by another antiparallel upper sheet of 5 β-strands [74]. The strands of the front and rear sheets can be composed of 5–7 residues, while the upper sheet consists of strands of 2–4 residues. Additionally, the β-strands are connected by loops [73,74]. The upper β-sheet is absent due to missing amino acids, resulting in disordered regions.

The front and rear sheets are parallel and are found at a distance of approximately 13 Å, as calculated by determining the distances between the centroids of the Cα atoms of each sheet in around 300 protein structures that contain the legume-type lectin fold [74]; this distance is required to form a hydrophobic residue cluster, where the aromatic side chains are positioned between the front and rear sheets, providing stability to the fold. As a general feature of many legume-type lectins, in their oligomeric structure, each monomer corresponds to a single canonical carbohydrate recognition domain (CRD), which is organized as dimers (Figure 7B) linked by salt bridges between the beta strands of the monomers [75].

**Figure 8:**
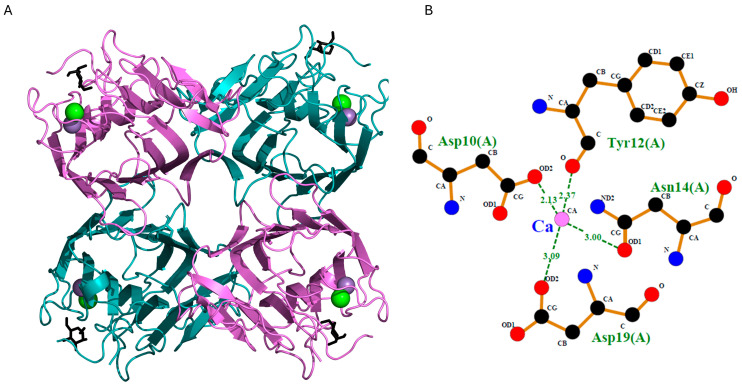
(**A**). Predicted tetrameric form of the GLL-I lectin. (**B**). Predicted interaction of Ca^2+^ (or Mn^2+^) ions in the lectin model. The residues involved in metal ion coordination—Asp10, Tyr12, Asn14, and Asp19 (all from chain A)—are highlighted. Green dashed lines represent coordination bonds, with distances indicated in Ångströms.

The monomeric form of the lectin GLL-I exists in equilibrium with its dimeric and tetrameric forms (Figure 7B and Figure 8A), similar to ConA. This equilibrium is influenced by pH and temperature conditions [76,77]. As previously mentioned, prolonged heating times are necessary during SDS-PAGE to dissociate the tetrameric form, as the detergent (SDS) alone is insufficient. Similarly, in the lectins from *Dioclea rostrata* (=*Macropsychanthus bicolor*) and *Dioclea grandiflora*, the presence of the conserved His51 residue may suggest its involvement in intradimeric interactions, likely of a van der Waals nature, with residues such as Thr194A, Thr49A, and Val64A, as well as interdimeric interactions with Val187, Val188, and Lys116, promoting the formation of the tetramer [75]. It is worth mentioning that this study did not identify residues Val187, Val188, and Thr194. Other studies have suggested that residues 117–123, located in a loop region, also play an important role in stabilizing the tetramer [78].

Each GLL-I subunit consists of 237 residues and contains two ion-binding sites, one for Ca^2+^ and another for Mn^2+^ (Figure 8B). This interaction is essential for the lectin to bind to carbohydrates [7,42,71,79]. The Ca^2+^ and Mn^2+^ ion-binding sites are conserved in lectins from the Diocleinae subtribe. The Ca^2+^ ion interacts with Asp10, Tyr12, Asn14, and Asp19, while the Mn^2+^ ion is coordinated by the residues Glu8, Asp10, and His24 [7,13,38]. Note that this lectin does not require the addition of Ca^2+^ and Mn^2+^ ions to exhibit its agglutination activity, nor does their addition enhance the interaction, as occurs with ConA. Experimental agglutination assays confirmed that GLL-I maintains activity without adding these divalent cations, indicating they are not essential for its carbohydrate-binding function. This suggests that in the structure of GLL-I, as in many legume lectins, the amino acids involved in binding Ca^2+^ (Tyr12, Asp10, Asp19, Asn14) (Figure 8B) or Mn^2+^ (Glu8, His24, Asp10, Asp19) are conserved. These interactions allow the formation of the three-dimensional structure of the carbohydrate-binding pocket in the lectin by stabilizing the unusual cis-peptide bond Ala207-Asp208. Interactions with the Ca^2+^ ion regulate the cis-trans isomerism of this bond [68].

#### Molecular Docking of the GLL-I Lectin

**Figure 9:**
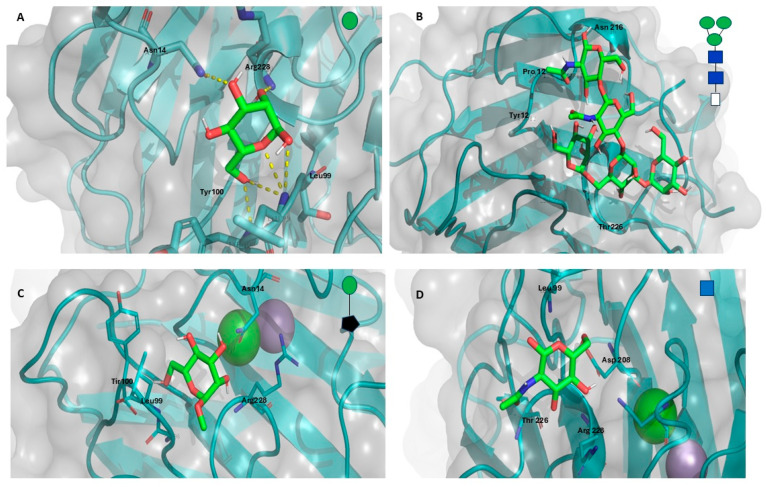
Molecular docking of the GLL-I lectin with some glycans. (**A**). αD-mannose. (**B**). DManα1-3[DManα1-6] DManβ1-4DGlcNAcβ1-4DGlcNAcβ-OH. (**C**). Methyl αD-mannopyranoside. (**D**). N-acetyl β-D-glucosamine. GLL-I is depicted as a cartoon (aquamarine green) overlaid with a gray molecular surface. Ligands are represented as sticks (carbon: green; oxygen: red; nitrogen: blue; hydrogen: white). The structure of each glycan is also presented in the upper right corner of each panel using the Symbol Nomenclature for Glycans (SNFG) [81].

The results of molecular docking indicate that the lectin GLL-I exhibits the highest interaction energy with the mannose-trisaccharide core (DManα1-3[DManα1-6]DManβ1-4DGlcNAcβ1-4DGlcNAcβ-OH) (Figure 9B) at −5.9 Kcal/mol, compared to βDGlcNAc (−5.2 Kcal/mol) (Figure 9D), methyl αD-mannopyranoside (−4.9 Kcal/mol) (Figure 9C), and αD-mannose (−4.8 Kcal/mol) (Figure 9A). These interaction energies demonstrate the preference of lectin GLL-I for complex carbohydrates, such as mannose-rich glycans. Type I lectins from the Dioclea tribe, including *Canavalia brasiliensis* lectin (ConBr), *Canavalia maritime* (=*C. rosea*) lectin (ConM), *Dioclea grandiflora* lectin (DGL), *Dioclea virgata* lectin (DVL), and *Cratylia floribunda* lectin (CFL), exhibit specificity towards mannose (Man), glucose (Glc), and their derivatives [80]. Other lectins, such as ConV, ConM, Cbol, and CGL, have demonstrated binding to dimannosides, validating the in silico docking results with the crystallography data of the complexes [13].

**Figure 10:**
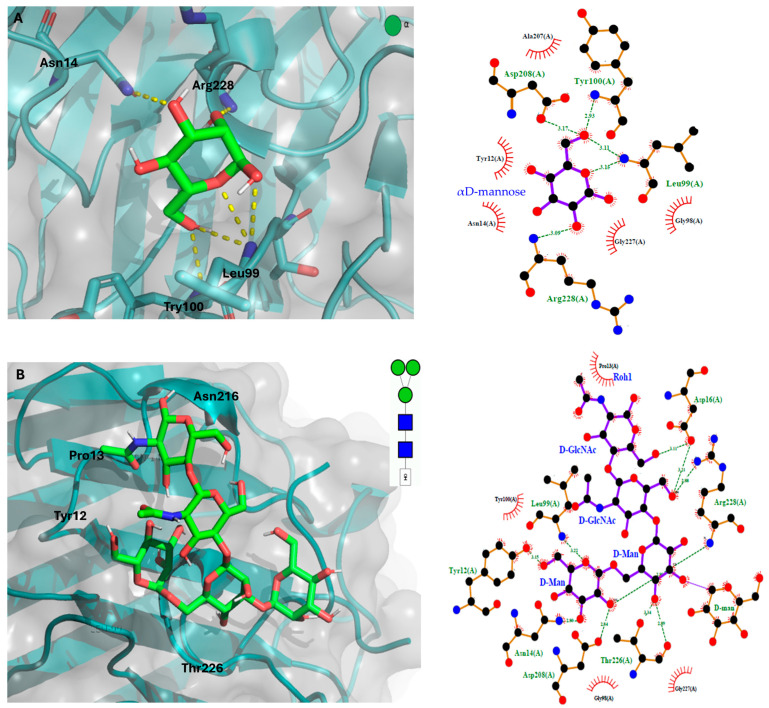
Three-dimensional and two-dimensional representations of the interaction between GLL-I and two ligands. (**A**). αD-mannose. (**B**). DManα1-3[DManα1-6] DManβ1-4DGlcNAcβ1-4DGlcNAcβ-OH. GLL-I is shown as a cartoon (aquamarine green) with a gray molecular surface. The ligands are sticks: carbon atoms in green, oxygen in red, nitrogen in blue, and hydrogen in white. The 2D diagram represents glycans as purple sticks, amino acid residues in yellow, and hydrogen bonds as green dashed lines.

The analysis of the recognition domain of GLL-I aligns with in silico studies reported by various authors, who have identified key residues involved in the interaction with different carbohydrates. These residues are conserved in this region compared to other type I lectins from Dioclea [7,38,63,82,83]. The amino acid residues of GLL-I, such as Tyr100, Asp208, and Leu99, interact with the OH6 of mannose. At the same time, Arg228 associates with the OH2 of the monosaccharide through hydrogen bond interactions (Figure 10A) (see Table S4).

Molecular docking analyses in lectins from other species, such as *Dioclea bicolor*, *Dioclea violacea* Mart. ex Benth, and *Dioclea sclerocarpa*, with a mannose residue, have demonstrated that the amino acid residues Arg228, Tyr100, Tyr12, Asn14, Leu99, Asn168, Asp208, and Gly226 are critical for the interaction, owing to the polar contacts that facilitate this binding [43,56,75,84]. In the case of the interaction between ConA and α-D-mannose, it has been described that the orientation of the hydroxyl groups at C3, C4, and C6 of the monosaccharide is crucial for effective interaction with the lectin [63]. This analysis reinforces the importance of conserved residues in lectin–carbohydrate interactions, suggesting common recognition mechanisms among type I lectins of the Diocleinae subtribe.

Recognition of the trimannoside involves hydrogen bonding between Asp16 and the O6 of β-D-GlcNAc-OH and D-GlcNAcβ1-4. Also, Arg228, Thr226, Asp14, Tyr12, Leu99, and Asp208 interact with D Man α1-6 and D Manα1-4, but not with D Manα1-3. The trimannoside resembles an open hand that fits into the carbohydrate-binding pocket of the lectin. In this configuration, the central mannose and the mannose linked by α1-6 establish hydrogen bonds with crucial residues. Conversely, the mannose linked by α1-3, corresponding to the outermost “finger,” does not form significant interactions. The last indicates that it is more exposed and plays a lesser role in stabilizing the binding process (Figure 10B). In the case of ConA lectin, α 1,6-linked mannose residue binds at the monosaccharide binding site, while the other two sugars interact within an extended cleft formed by the residues Tyr-12, Pro-13, Asn-14, Thr-15, and Asp-16. Hydrogen bonds are established between the protein and all three sugar residues. A conserved water molecule is also crucial in anchoring the reducing sugar unit to the protein [85]. Lectins with similar specificities often interact with carbohydrates differently, resulting in varying affinities. In this instance, GLL-I likely uses a unique recognition mechanism compared to type I lectins from the Diocleinae subtribe. However, X-ray diffraction studies are necessary to better understand how GLL-I interacts with this type of oligosaccharide.

### 2.6. Cytotoxicity of Lectin GLL-I Against the Cf-203 Cell Line

**Figure 11:**
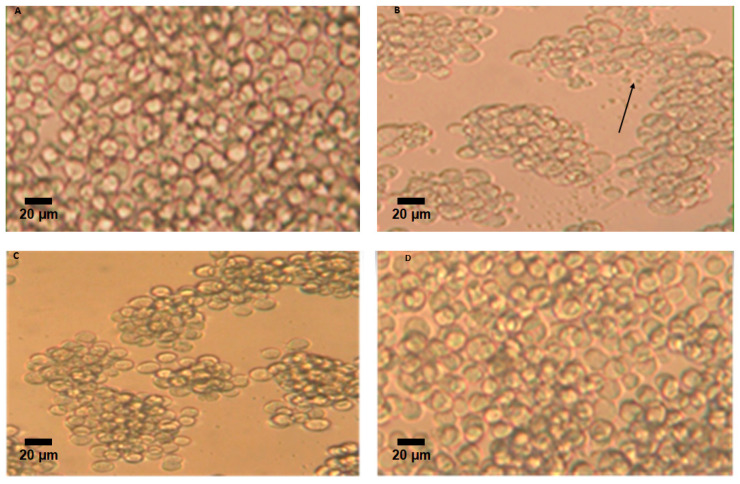
Effect of GLL-I on CF-203 cells incubated with the lectin for 4 days at 27 °C. (**A**) Control. (**B**) Cells treated with 10 μM GLL-I. The arrow indicates cellular debris. (**C**) Cells treated with 3 μM. (**D**) Cells treated with 0.03 μM. Scale bar: 20 μm. Cell viability is expressed as percentage of change relative to control and presented and compared in Figure 12. There were statistically significant differences between the control group and the 10 μM treatment (*p* < 0.0001), as well as between the control and the 0.03 μM treatment (*p* < 0.01). Moreover, significant differences in cell viable percentage were found between the 10 μM treatment and the 3, 1, 0.3, and 0.03 μM concentrations (*p* < 0.05, *p* < 0.001, *p* < 0.005, and *p* < 0.005, respectively).

**Figure 12:**
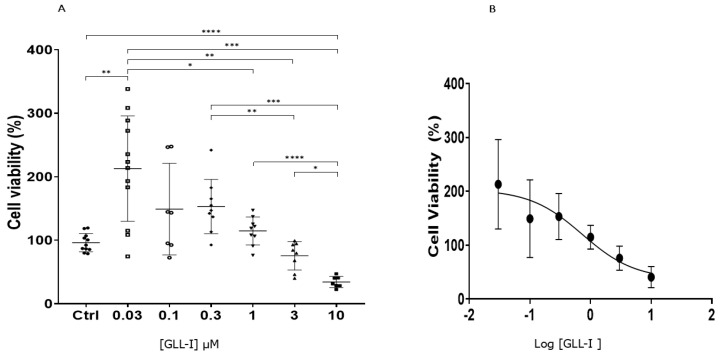
Effect of lectin GLL-I on the viability of CF203 cells. (**A**). Percentage of cell viability after 4 days of treatment at 27 °C with increasing concentrations of GLL-I. Statistically significant differences are indicated: * *p* < 0.05; ** *p* < 0.01; *** *p* < 0.005; **** *p* < 0.001. (**B**) Dose–response curve representing the effect of GLL-I on CF203 cell viability. Data are presented as mean ± standard deviation from three independent experiments and four measurements at each protein concentration and the means ± SD were analyzed using a Tukey–Kramer post hoc test (*p* = 0.05) using GraphPad Prism version 8 (GraphPad Software, San Diego, CA, USA, www.graphpad.com). Additionally, dose–response curves were generated to determine the IC50 concentration.

GLL-I, at a concentration of 10 μM, demonstrated a cytotoxic effect on CF-203 cells derived from the midgut of insects after four days of exposure. The observations included cellular shrinkage, increased cellular debris, and decreased viable cells when GLL-I was added (Figure 11B and Figure 12A) compared to the control group (Figure 11A and Figure 12A). Cell viability decreased by 52% and 59.4% at concentrations of 3 μM and 10 μM of GLL-I, respectively, suggesting that this lectin may have insecticidal properties and could be considered a potential agent for pest control. Some cellular damage was also observed in cells treated with 3 μM of GLL-I, although the reduction in cell numbers was less pronounced (Figure 11C and Figure 12A). In contrast, the cell count increased at a concentration of 0.03 μM (Figure 11D and Figure 12A), with a viability percentage exceeding 150%. The calculated IC50 for GLL-I was 9.528 nM (571 μg), demonstrating a dose-dependent effect of GLL-I (Figure 12B).

Significant differences were observed between the concentrations 10 μM and other concentrations. In this case, only 10 μM provoked significant decrease in cell viability (Figure 12A). It is important to say that the percentage of decrease relative to the control is not significant for 3 uM (75.71%). Therefore, significant differences were observed between 3 μM and 0.3 μM, and 3 μM and 0.03 μM (*p* < 0.005 and *p* < 0.01, respectively). However, there were no significant differences found between 3 μM and 1 μM, nor between 3 μM and 0.1 μM. Notably, a significant difference was identified between the concentration of 1 μM and 0.03 μM (*p* < 0.05). The percentages of cell viability for the concentrations of 3 μM, 1 μM, 0.3 μM, 0.1 μM, and 0.03 μM were 75.71%, 114.76%, 145.53%, 131.18%, and 212.95%, respectively (Figure 12A).

The dose–response curve (Figure 12B) reveals a logarithmic, inverse relationship between GLL-I concentration and CF203 cell viability. This trend indicates a dose-dependent effect, characteristic of compounds with cytotoxic or antiproliferative activity. The active concentration range appears to lie between 0.1 and 10 μM, suggesting that this interval may be suitable for estimating an approximate IC_50_ value (calculated according to the equation derived from Figure 12B) in future analyses.

Research has shown that exposure to the agglutinin from *Rhizoctonia solani* J.G. Kuhn (RSA), isolated from mycelium, significantly decreased the viability of the CF203 cell line by 86 ± 2% at a concentration of 0.7 μM. At a lower concentration of 0.3 μM, RSA exhibited a toxicity level of 41 ± 8% [86]. In contrast, GLL-I at the same concentration increased cell viability by 145% (Figure 12A). RSA is a type of ricin lectin composed of a homodimer formed by two non-covalently associated monomers of 15.5 kDa [86]. It is classified as a basic protein (pI > 9) and shows specificity towards Gal/GalNAc residues. The hydroxyl groups located at positions C3′, C4′, and C6′ of the pyranose ring are crucial for interacting with simple sugars [87].

Furthermore, RSA preferentially recognizes human type A erythrocytes and is inhibited by N-acetylgalactosamine (GalNAc) at a concentration of 1 mM [87]. Regarding the specificity of GLL-I, D-mannose partially inhibited its activity, suggesting that both lectins may recognize different cellular targets to induce a cytotoxic effect. This is evident as treatment with the SNA-I lectin from *Sambucus nigra* L. at concentrations above 0.04 μM causes a decrease of 90–100% in cell viability [88], by recognizing the terminal sialic acid, preferentially in the α2-6 configuration (Neu5Ac α2-6 Gal). Similarly, the SNA-II lectin, at concentrations above 0.017 μM, also reduces cell viability [89], by recognizing the terminal sialic acid, preferentially in the α2-6 configuration (Neu5Ac α2-6 Gal). Moreover, the SNA-II lectin, at concentrations above 0.017 μM, reduces cell viability, although it recognizes mannose residues [89,90].

Lectins GLL-I, RSA, SNA-I, and SNA-II exhibit a dose-dependent cytotoxic effect on the CF203 cell [86,89,91]. The cytotoxic effect of the lectin GLL-I at a concentration of 10 μM in CF203 cells (Figure 12B) is likely due to the induction of cell death by apoptosis, as reported for the lectins SNA-I and SNA-II in CF203 cells. These lectins also caused a loss of cell viability with typical apoptotic features, such as cell shrinkage, membrane blebbing, nuclear condensation, and DNA fragmentation. A caspase-dependent pathway mediates this cytotoxic effect and requires the carbohydrate-binding activity of the lectins [89,92]. In addition, other lectins have also demonstrated cytotoxicity in various cell lines, such as human monocytes (U937), bovine endothelial cells, and canine kidney cells (MDCK) [89,92,93].

Lectin GLL-I might exhibit cytotoxicity through a mechanism similar to that described for ConBr, as it promoted cell death in CF-203 cells at the highest concentration tested. ConBr, a lectin known for its antiglioma activity, induces cell death in C6 glioma cells at elevated concentrations. Its cytotoxicity modulates MAPK and Akt signaling pathways, which promote autophagy and caspase-8-dependent cell death. This effect is partially dependent on the integrity of the three-dimensional structure of the carbohydrate recognition domain (CRD) [94]. Given that lectins share structural characteristics in their carbohydrate-binding capabilities, it is possible that GLL-I also induces cytotoxicity by activating similar pathways, suggesting the potential involvement of apoptotic or autophagic routes in its action on CF-203 cells. This type of lectin-mediated cytotoxic mechanism has been observed in other cellular models, reinforcing the hypothesis that lectins may act as key modulators in regulating cell death [89,92,93].

Interestingly, it was observed that GLL-I promoted cell proliferation at a concentration of 0.03 μM, showing statistically significant differences from the control (Figure 12A). Some plant lectins have demonstrated mitogenic effects in various cell types. For example, the lectin ConBr induced the proliferation of murine splenocytes at concentrations of 10, 5, and 2.5 μg/mL, with the maximum proliferation index observed at 10 μg/mL [95]. Similarly, the lectin from *Artocarpus lingnanensis* Merr (ALL) stimulated the proliferation of human T lymphocytes [96]. Additionally, it was reported that a zebrafish liver cell line (ZFL) exposed to 10 μg/mL of *Arachis hypogaea* L. lectin (PNA) for 48 h showed a 52.4 ± % increase in cell proliferation, with no observable effects on apoptosis [97]. This proliferative effect of PNA correlated with an increase in the levels of cell cycle progression markers and anti-apoptotic proteins. Likewise, PNA stimulated the proliferation of cancer cell lines and increased the expression of glucose-6-phosphate dehydrogenase, a key enzyme in the pentose phosphate pathway, which is directly related to cell proliferation [98].

Lectin GLL-I, at a concentration of 0.03 μM, may act similarly to other lectins from the Subtribe Diocleinae by binding to growth factors in CF203 cells. This would explain the increase in cell proliferation observed in this study. This mechanism, mediated by interaction with cell surface receptors, can activate intracellular signaling pathways that promote cell proliferation. The interaction between lectins and membrane glycoprotein receptors has been described as a key factor in regulating processes such as proliferation, differentiation, and activation of immune cells, as seen with lectins such as PHA from *Phaseolus vulgaris* L., PSA from *Pisum sativum* L., and ConA, which recognize Glc/Man residues [95,99,100,101].

The ability of GLL-I to stimulate cell proliferation, along with its cytotoxic effect at higher concentrations, suggests that this lectin has a dual impact on CF203 cells that is concentration-dependent. This behavior may be related to the differential activation of cellular signaling pathways or the affinity of GLL-I for different carbohydrate structures that contain mannose residues on cell surface receptors.

### 2.7. Binding of GLL-I Lectin to the Digestive Tract of Spodoptera Frugiperda

**Figure 13:**
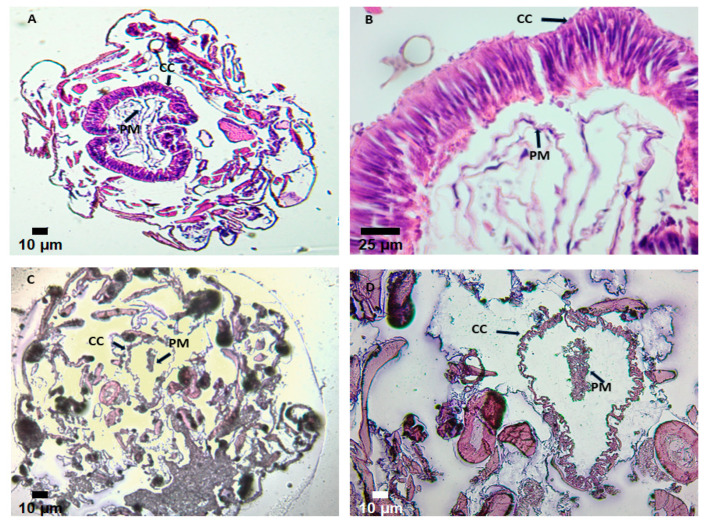
Cross-section of the midgut of fourth-instar *Spodoptera frugiperda* larvae. (**A**,**B**) control larvae. (**A**): General view of the midgut cross-section showing columnar cells (CC) and the peritrophic membrane (PM). Scale bar: 10 μm. (**B**): Magnified view of a midgut section highlighting the columnar cells (CC) and the peritrophic membrane (PM). Scale bar: 25 μm. (**C**,**D**) GLL-I treated larvae. (**C**): Detailed view of the columnar cells and peritrophic membrane in a midgut cross-section. Scale bar: 10 μm. (**D**): Midgut cross-section of *S. frugiperda* after feeding larvae with biotinylated GLL-I. Scale bar: 10 μm.

Histological changes were observed in the midgut of *Spodoptera frugiperda* Walker (Noctuidae) larvae after 24 h of control (Figure 13A,B) and lectin ingestion (Figure 13C,D). The lectin is bound to the peritrophic membrane (PM), altering its structure with significant retraction compared to the apical surface of the columnar cells (CC). Additionally, irregular shapes were evident in epithelial cells and adjacent tissues (Figure 13C,D). In contrast, Figure 13A,B show cross-sections of the controls (magnifications of 4× and 40×, respectively), stained with hematoxylin-eosin, obtained from larvae fed a lectin-free diet. A well-structured epithelium is observed in these sections, composed of elongated columnar cells, goblet cells, and intact PM. The cell nuclei exhibit an elongated shape, located in the medial-basal region of the cells (Figure 13B).

These results suggest that the ingestion of GLL-I induces significant morphological alterations in the midgut of fourth instar *S. frugiperda* larvae, affecting the integrity of the peritrophic membrane (PM) and the intestinal epithelium, thereby reducing their structural stability, which is essential for the growth and development of the insects [102]. The binding of GLL-I increases the mortality of *S. frugiperda* larvae, reduces pupal weight, and negatively impacts the emergence of adults similarly as has been reported for a peritrophin [103]. The PM is a key protective barrier in the intestines of insects, and its disruption could compromise the digestion and absorption of nutrients and protection against pathogens and toxins. The modifications observed in epithelial cells, such as the loss of their regular structure and the displacement of nuclei, may be related to the disruption of intestinal homeostasis, potentially affecting cell viability and causing cytotoxic effects. Similarly, the lectin from *Moringa oleifera* (WSMoL) has also shown adverse effects in *Anagasta kuehniella*, reducing larval weight through the disruption of the PM. Furthermore, it has been reported that WSMoL binds to chitin and may induce apoptosis in intestinal cells [104].

In vivo experiments were previously carried out at a concentration of 1 μg lectin/g diet of fourth-instar larvae, showing decreased survival and decreased weight of the larvae. However, due to the limited amount of protein we obtained, it was not possible to perform adequate replications to statistically conclude on the effect generated. It was only possible to perform one assay in triplicate at a single lectin concentration, but the results obtained there were the basis for designing the histochemistry tests whose results are shown and discussed in this paper. Future research includes exploring practical applications of lectin GLL-I as a potential biological insecticidal agent and its effects on other insect species, as well as assessing its viability in agricultural pest control. Furthermore, it is essential to delve into the molecular mechanisms underlying the observed toxicity and/or proliferative effects to better understand the lectin’s specific interactions with the glycans on the surface of insect intestinal cells. These studies could open new avenues for developing more effective and sustainable pest control agents.

## 3. Materials and Methods

### 3.1. Collection of Plant Material

The plants and seeds of *Galactia lindenii* were collected near the Fúquene Lagoon, located at coordinates (5.474034286259336, −73.77022153699131) in Cundinamarca, Colombia. The specimens were identified at the Institute of Natural Sciences (ICN) at the Universidad Nacional de Colombia in Bogotá. Vouchers for the specimens include Fernández-Alonso 15115 (CB/COL55551) and Z. Casas 1 (CB/COL580116). Both images can be verified in the virtual COL collection vouchers COL 15115 and COL 580116. This study is conducted under the genetic resource access contract number 246, granted by the Ministry of Environment and Sustainable Development (MADS).

### 3.2. Extraction and Purification of Galactia Lindenii Type I Lectin (GLL-I)

The seeds were separated from the plant material and ground into flour to prepare a soluble protein extract. Five grams of the flour were mixed with 50 mL of acetate-acetic buffer at pH 5.0, which consisted of 100 mM sodium acetate and 5 mM thiourea. In parallel, another extract was prepared using 50 mL of PBS at pH 7.25 (20 mM Na_2_HPO_4_, 20 mM NaH_2_PO_4_, and 150 mM NaCl), also with 5 mM thiourea. Both mixtures were incubated for 8 h at 4 °C with continuous shaking. Following incubation, the mixtures were centrifuged at 12,000× *g* for 45 min at 4 °C. This extraction procedure was repeated three times using the same plant material. The three supernatants were combined and concentrated by ultrafiltration using an Amicon membrane with a cut-off of 10 kDa. The final extract was stored at −20 °C for subsequent assays.

Three purification methods were carried out on the concentrate to obtain the lectin. In the first method, 15 mL of the concentrated pool was subjected to discontinuous ion-exchange chromatography on a DEAE-Sephadex A-50 column (12 cm × 2 cm), which was equilibrated with PBS (Na_2_HPO_4_ 20 mM, NaH_2_PO_4_ 20 mM, NaCl 150 mM). The unbound fraction (designated as DEAE-FNR) was eluted with the equilibration buffer, while the bound fraction (DEAE-FR) was eluted by increasing the ionic strength with 0.5 M NaCl. Subsequently, the bound fraction (DEAE-FR) was dialyzed against 20 mM NH_4_HCO_3_ and concentrated using an Amicon^®^ (Merck KGaA, Darmstadt, Germany) filter with a molecular weight cut-off of 10 kDa. Secondly, this fraction was loaded onto a Sephacryl S-200 column (10 cm × 2 cm) (GE Healthcare Handbook, 2010), which was equilibrated with PBS pH 7.2. The unbound fraction (Sephacryl-FNR) was eluted with PBS, while the bound fraction (Sephacryl-FR), which contains GLL-I, was eluted using 0.2 M glucose (Glc) in PBS.

The third step involved loading 2 mL of the concentrated fraction (DEAE-FR) equilibrated in 20 mM ammonium bicarbonate (NH_4_HCO_3_) containing 1 M sodium chloride (NaCl) onto a Hiprep 16/60 Sephacryl S200 HR column. Elution was performed at a flow rate of 0.05 mL per minute. Before this, the column was washed with two volumes (240 mL) of degassed, filtered MQ water at a flow rate of 0.05 mL per minute, and then equilibrated with 20 mM NH_4_HCO_3_ and 1 M NaCl solution. In the final purification step, the active fraction was dialyzed against 20 mM ammonium bicarbonate (NH_4_HCO_3_), then lyophilized and stored at 4 °C for future assays.

The protein concentration was measured using the Bicinchoninic Acid (BCA) assay and the micro-Kjeldahl method [51,105]. SDS-PAGE was conducted following the protocol established by Laemmli in 1970 [106]. Hemagglutination activity was assessed using human O+ and A+ red blood cells (RBCs) [107], and specific titers were determined at each stage of purification. The specific titer is the agglutination titer divided by the protein concentration (mg/mL). The minimum agglutinating concentration (MAC) is the lowest lectin concentration at which visible agglutination occurs, measured on a qualitative scale from 0 to +4.

### 3.3. Hemagglutination Inhibition by Different Carbohydrates

For the hemagglutination assays, a 15 µL solution of lectin (at a concentration of 0.125 mg/mL) was combined with 50 µL of carbohydrate solutions (at 0.3 M) that included Glucose (Glc), Mannose (Man), Lactose (Lac), Galactose (Gal), Fucose (Fuc), N-acetylgalactosamine (GalNAc), 1-O-methyl-β-galactopyranoside, 1-O-methyl-β-glucopyranoside, β-methyl D-glucose, N-acetyl-α-D-glucosamine (GlcNAc), α-methyl O-mannoside, and D-galacturonic acid (GlcA).

Additionally, 50 µL of each of the following solutions (at 37 mM) was used: p-Nitrophenyl-β-D-glucopyranoside, p-Nitrophenyl-α-D-glucopyranoside, p-Nitrophenyl-β-D-glucosamine, p-Nitrophenyl-N-acetyl-α-D-glucosamine, p-Nitrophenyl-β-D-mannopyranoside,p-Nitrophenyl-β-D-galactopyranoside,p-Nitrophenyl-N-acetyl-β-D-galactosamine, and p-Nitrophenyl-N-acetyl-β-D-galactopyranoside. After mixing the lectin and carbohydrate solutions, they were incubated for 30 min. Then, 50 µL of a 2% suspension of A+ erythrocytes was added, and the mixture was incubated for 2 h at room temperature. After the incubation period, agglutination was evaluated using a qualitative scale from 0 to +4, confirmed by light microscopy using 10× and 20× magnification.

Serial dilutions were performed to determine the minimum inhibitory concentration (MIC) of carbohydrates that inhibited the lectin activity. Each dilution used 50 µL of carbohydrate solution and 50 µL of the lectin solution (0.125 mg/mL). These mixtures were incubated for 30 min, after which 50 µL of the A+ erythrocyte suspension was added. The assays were then left to react for one hour at room temperature, and agglutination was evaluated at this time.

### 3.4. Isoelectric Focusing and Glycosylation

The isoelectric point (pI) was determined using a mixture of ampholytes with a pH range of 3.5–10 (Protein Mixture^®^ from GE Healthcare, Sunnyvale, CA, USA) under non-denaturing conditions, following the methodology described by Bollag and Edelstein (1994) [108]. Calibration curves were established using standards within a pH range of 3 to 10 (Pharmacia, now part of Cytiva Marlborough, Marlborough, MA, USA). Myoglobin at 5 μg/μL was used as the running control. The running solutions consisted of 0.1 M phosphoric acid at the anode and 0.1 M NaOH at the cathode.

The isolated protein was separated using an SDS-PAGE gel and transferred onto a nitrocellulose membrane to identify glycoproteins qualitatively. Carbohydrate oxidation was performed with 30 mM sodium meta-periodate, followed by a reaction that linked vicinal aldehydes with hydrazide-biotin. Detection was conducted using the streptavidin-peroxidase system, with ovalbumin glycoprotein at 5 μg/μL as the assay control.

### 3.5. Amino Acid Sequence

#### 3.5.1. Sample for Peptide Mapping

The lectin was solubilized in 0.1% sodium dodecyl sulfate (SDS) with 0.05 M dithiothreitol (DTT) and then heated at 60 °C for 30 min. After this, 55 mM iodoacetamide was added, and the mixture was incubated in the dark at room temperature for 30 min. The protein was then precipitated using 10% trichloroacetic acid (TCA) and placed on ice for 15 min. Afterward, acetone was added, and the mixture was centrifuged at 16,000 relative centrifugal force (rcf) for 15 min at 4 °C. The resulting pellet was resuspended in 50 mM Tris-HCl, pH 8.0, and digestion was performed using trypsin (Sigma Aldrich, Burlington, MA, USA, proteomic grade) at an enzyme-to-sample ratio of 1:10. The digests were stored at 7 °C for analysis by liquid chromatography–mass spectrometry (LC-MS/MS) and peptide mapping [109].

Using the de novo peptide sequences, the Decrease Redundancy tool from EXPASY [67] was employed to reduce redundancy among peptides with 90% or greater identity. The resulting peptides were compared to legume lectin sequences using blastP from BLAST [110], available at NCBI, and utilizing algorithms designed for short sequences (https://blast.ncbi.nlm.nih.gov/Blast.cgi?PROGRAM=blastp&PAGE_TYPE=BlastSearch&BLAST_SPEC=&LINK_LOC=blasttab&LAST_PAGE=blastp, accessed on 16 September 2025) to facilitate peptide assembly.

The sequence alignment was performed using the previously selected peptides, and a comparison was made in blastP against the type I lectin sequence from *Canavalia ensiformis* (ConA) (PDB 1APN_A) for the peptides that aligned with regions of ConA; a complete alignment was carried out, selecting the peptides that aligned with the lowest E-value, the highest identity percentage, and the highest sequence coverage.

#### 3.5.2. Multiple Sequence Alignments of GLL-I with Lectins from the Sutribe Diolceinae

A multiple sequence alignment of GLL-I was performed using BLAST against the sequences of *Canavalia ensiformis* lectin (ConA) (PDB 1APN_A) and GLL-II (GenBank: WAB55723.1). Additionally, a multiple sequence alignment was conducted using T-Coffee with the lectins ConA (PDB 1APN_A), *Canavalia brasiliensis* (ConBr) (sp P55915.1), *Canavalia maritima* (=*Canavalia rosea*) (ConM) (PDB 2OW4_A), *Canavalia boliviana* (ConBol) (PDB 4K20_B), *Canavalia grandiflora* (ConGF) (PDB 4L8Q_A), *Cymbosema roseum* (CRLI) (PDB 3A0K_G), *Dioclea lasiocarpa* (DLL) (PDB 5UUY_A), *Dioclea rostrata* (=Macropsychanthus bicolor) (DRL) (PDB 2ZBJ_A), and *Dioclea grandiflora* (DGL) (PDB 1DGL_B). The alignment was visualized using ESPript 3.0. [111]. The lectins with which GLL-I had the highest coverage and the lowest *p*-value were selected.

#### 3.5.3. Molecular Docking Studies of the GLL-I

Modeling was performed based on the primary structure using the SWISS-MODEL (https://swissmodel.expasy.org/, accessed on 16 September 2025), and the quality of the model was evaluated using the PBDSUM server (https://www.ebi.ac.uk/thornton-srv/databases/pdbsum/, accessed on 16 September 2025). The interaction analysis of GLL-I with specific carbohydrates was carried out through molecular docking, and the resolved model files were prepared using AutoDockTools 1.5.6. [112]. Molecular docking was performed using AutoDock Vina [113], defining GLL-I as the receptor and the following carbohydrates as ligands: trimannoside DMan α1-3 [DManα1-6] DManβ1-4 DGlcNAcβ1-4 DGlcNAcβ-OH, βD GlcNAc, Methyl αD manopyranoside (Methyl αD Man), αDManose (αDMan), L-Fucose, and αDGlucose (αDGlc), carbohydrates present in insect cells. The structures of the ligands were downloaded from Glycam (https://glycam.org/, accessed on 16 September 2025) and PubChem (https://pubchem.ncbi.nlm.nih.gov/docs/compounds/, accessed on 16 September 2025). The docking procedure treated the protein as rigid, while the ligands were flexible. The binding site was defined in the CDR region of the template lectin with the following coordinates: center x = 34; center y = 56; center z = 40; size x = 19.192; size y = 0.383; size z = 22.298. Interactions were visualized using PyMOL, and tables were created by selecting the most negative values as indicators of the most favorable binding interactions.

### 3.6. Cytotoxicity Assays of GLL-I Against the CF-203 Cell

The cell line CF203 provided by the Laboratory of Agrozoology of Ghent University [86], obtained from the midgut of the moth *Choristoneura fumiferana* Clemens (Tortricidae), in the logarithmic growth phase (4 days post-subculture), was suspended twice in Insect-Xpress medium (Bio-Whittaker-Cambrex Bioscience, Baltimore MA, USA) supplemented with 2.5% FBS (fetal bovine serum) (Sigma Aldrich, Burlington, MA, USA). GLL-I in 0.9% NaCl was added in a 1:10 ratio to achieve final concentrations of 10, 3, 1.0, 0.3, 0.1, and 0.03 µM. Subsequently, 100 µL aliquots of the cell suspension with the lectin and controls with 0.9% NaCl were transferred to 96-well flat-bottom transparent plates and incubated for 4 days.

After this period, the cells were transferred to black 96-well plates and mixed with the fluorescent reagent PrestoBlue^®^ (Invitrogen, Carlsbad, CA, USA) in a 1:10 ratio to assess cell viability. After 20 min of incubation at 27 °C, fluorescence was measured using a Tecan plate reader (Tecan, Männedorf, Switzerland) (excitation at 560 nm and emission at 600 nm) [114]. The proportion of viable cells was calculated based on serial dilutions of control cells treated with NaCl 0.9%. Three independent experiments were performed with the lectin, and four measurements were taken at each protein concentration for each experiment. Viability percentage data were expressed as mean ± SD for each treatment and control. Significant differences between treatments were analyzed using analysis of variance (ANOVA), and the means ± SD were analyzed using a Tukey–Kramer post hoc test (*p* = 0.05) using GraphPad Prism version 8 (GraphPad Software, San Diego, CA, USA, www.graphpad.com). Additionally, dose–response curves were generated to determine the IC50 concentration (according to the equation of established graphic relationship).

### 3.7. Binding of the GLL-I Lectin to the Digestive Tract of Spodoptera

To determine the binding to the digestive tract of *S. frugiperda*, GLL-I was biotinylated using sulfo-N-hydroxysuccinimide ester of biotin (sulfo-NHS-LC-biotin, Sigma) [115]. A total of 0.5 mg of sulfo-NHS-LC-biotin was mixed with 0.5 mg of GLL-I in PBS (w/w) and incubated for 12 h at room temperature. Subsequently, biotinylated GLL-I was dialyzed against 20 mM NH_4_HCO_3_, lyophilized, and stored at 4 °C.

Fourth, instar larvae of Spodoptera frugiperda were fed for 24 h an artificial diet that contained biotinylated GLL-I at 0.1 mg/mL. Controls were performed with larvae fed on the artificial diet without the addition of lectin. The treated larvae and controls were fixed in Carnoy’s solution (ethanol–chloroform–acetic acid, 6:3:1) for 24 h, then dehydrated in 70%, 95%, and 100% ethanol for 30 min each. The larval bodies were placed in butanol for 24 h, followed by embedding in paraffin (1:1) for 12 h. Subsequently, 6 µm thick sections were made using a microtome [86].

For histochemistry, the slides were deparaffinized for 20 min at 60 °C. They were then rehydrated using xylene, absolute ethanol, 96% ethanol, and 70% ethanol, exposing them to each solvent for 4 min. After rehydration, the slides were washed with distilled water and PBS-Tween (0.1%) adjusted to a pH of 7.2–7.4. Permeabilization was performed with 0.1% Triton X-100 in PBS for 30 min at room temperature.

Then, a citrate buffer at pH 6.0 (Novocastra ref RE7113; Leica microsystems, Wetzlar, Germany) was added for 20 min at room temperature. Endogenous peroxidase was inactivated using a solution of 10% methanol and 0.3% hydrogen peroxide in PBS for 30 min. Blocking was performed with 200 μL of 10% fetal bovine serum (FBS) for 1 h at 37 °C. Next, 100 μL of a peroxidase-avidin solution (1:500) in PBS-FBS (10%) was applied for 1 h at room temperature.

The slides were developed for one minute with a 1% DAB solution in Tris-HCl (50 mM), pH 7.3, adding 5 μL of 30% H_2_O_2_. Three washes with PBS-Tween (0.1%) pH 7.2–7.4 were performed at each process step. Counterstaining was carried out using Harris hematoxylin for 1 min, followed by progressive dehydration with 70%, 90%, and 95% ethanol for 4 min each, ending with xylene for 4 min in three changes.

The slides were finally mounted with Cytoseal. Images were acquired using a Carl Zeiss Primo Star microscope (Zeiss, Oberkochen, Germany) with an Axiocam Erc 5S Zeiss camera (P95-C ½” 0.5 × 4155500-1811-000).

## 4. Conclusions

This study successfully purified a novel *Galactia lindenii* Type I lectin (GLL-I) using ion exchange and affinity chromatography. SDS-PAGE analysis revealed two distinct bands corresponding to the monomer (30 kDa) and dimer (60 kDa), with an isoelectric point (pI) of 6.15, confirming its glycoprotein nature. The most potent inhibitor of GLL-I agglutination activity was p-nitrophenyl-β-D-mannopyranoside. Molecular docking analysis indicated that GLL-I exhibits the highest affinity for a high-mannose tetrasaccharide (DManα1-3[DManα1-6] DManβ1-4DGlcNAcβ1-4DGlcNAcβ-OH). The proposed sequence covered 79% of the complete protein, sharing 188 of the 237 amino acids present in ConA lectin. Sequence analysis demonstrated a high degree of similarity to lectins from the Canavalia genus and lower similarity to those from the *Dioclea* genus. Furthermore, structural modeling confirmed that GLL-I adopts a canonical legume lectin fold.

Functionally, GLL-I exhibited dual biological effects in CF203 cells, inducing cytotoxicity at 10 µM after four days of exposure while promoting cell proliferation at a lower concentration (0.03 µM). Histochemical analysis further demonstrated that GLL-I binds to the midgut cells of fourth-instar *Spodoptera frugiperda* larvae, aligning with the behavior observed in other lectins with insecticidal properties. These findings establish GLL-I as a structurally and functionally distinct legume lectin with promising biotechnological applications. Its selective cytotoxicity and binding specificity position it as a potential candidate for development in pest control strategies and as a tool for targeting glycan structures in biomedical research.

## Figures and Tables

**Table 2:** MIC values of carbohydrates inhibiting GLL-I activity.

Carbohydrate	Initial Concentration (mM)	Minimum Inhibitory Concentration (MIC) (mM)	Relative Inhibitory Activity **
D-Manose	300	150	1
p-Nitrophenyl-α-D-mannopyranoside	37	37	4
p-Nitrophenyl-β-D-manopyranoside	37	9.5	16

** Relative inhibitory activity: calculated as the ratio between the MIC of mannose (Man) and the MIC of the evaluated monosaccharide.

## Data Availability

Data are available upon reasonable request.

## Supplementary Materials

The following supporting information can be downloaded at https://www.mdpi.com/article/10.3390/ijms262110359/s1. References [116,117,118,119] are cited in the Supplementary Materials.

## Abbreviations

The following abbreviations are used in this manuscript:

GLL-I	Type I Lectin from Galactia lindenii
GLL-II	Type II Lectin from Galactia lindenii
Glc	Glucose
Man	Mannose
kDa	Kilodaltons
CF203	Midgut cell line of *Choristoneura fumiferana*
GalNac	N-acetylgalactosamine
ConBr	Lectin from *Canavalia brasiliensis*
DGL	*Dioclea grandiflora* lectin
DguiL	*Dioclea guianensis* lectin
ConA	Lectin from *Canavalia ensiformis*
DEAE	Diethylaminomethyl
FR S200	Sephacryl S200 retained fraction
DTT	Dithiothreitol
ConGF	Lectin from *Canavalia grandiflora*
DrfL	Lectin from *Dioclea reflexa*
DBL	*Dioclea bicolor* lectin
MCI	Minimum inhibitory concentration
CRL-I	*Cymbosema roseum* lectin
pI	Isoelectric point
DSL-I	*Dioclea sericea* lectin
ConM	Lectin from *Canavalia maritima (=Canavalia rosea)*
ConBol	Lectin from *Canavalia boliviana*
DLL	*Dioclea lasiocarpa* lectin
DRL	*Dioclea rostrata (=Macropsychanthus bicolor)* lectin
DVL	*Dioclea virgata* lectin
CFL	*Cratylia floribunda* lectin
RSA	Agglutinin from *Rhizoctonia solani*
SNA-I	Lectin I from *Sambucus nigra*
SNA-II	Lectin II from *Sambucus nigra*
CRD	Carbohydrate recognition domain
ALL	*Artocarpus lingnanensis*
ZFL	Zebrafish liver cell line
PNA	*Arachis hypogaea*
PHA	*Phaseolus vulgaris* lectin
PSA	*Pisum sativum* lectin
PM	Peritrophic membrane
CC	Columnar cells
WSMoL	Lectin from *Moringa oleifera*
FNR −	Unretained fraction
FNR +	Retained fraction

## References

[1] Chettri D., Boro M., Sarkar L., Verma A.K. (2021). Lectins: Biological Significance to Biotechnological Application. Carbohydr. Res..

[2] Konozy E.H.E., Osman M.E.M. (2022). Plant Lectin: A Promising Future Anti-Tumor Drug. Biochimie.

[3] Konozy E., Osman M., Dirar A. (2022). Plant Lectins as Potent Anti-Coronaviruses, Anti-Inflammatory, Antinociceptive and Antiulcer Agents. Saudi J. Biol. Sci..

[4] Tsaneva M., Van Damme E.J.M. (2020). 130 Years of Plant Lectin Research. Glycoconj. J..

[5] Fujimoto Z., Tateno H., Hirabayashi J. (2014). Lectin Structures: Classification Based on the 3-D Structures. Methods Mol. Biol..

[6] Prabu M.M., Suguna K., Vijayan M. (1999). Variability in Quaternary Association of Proteins with the Same Tertiary Fold: A Case Study and Rationalization Involving Legume Lectins. Proteins.

[7] Cavada B.S., Pinto-Junior V.R., Osterne V.J.S., Nascimento K.S. (2018). ConA-like Lectins: High Similarity Proteins as Models to Study Structure/Biological Activities Relationships. Int. J. Mol. Sci..

[8] Queiroz L.P., Oliveira A.C.S., Snak C. (2020). Disentangling the Taxonomy of the Galactia-Camptosema-Collaea Complex with New Generic Circumscriptions in the Galactia Clade (Leguminosae, Diocleae). Neodiversity.

[9] Queiroz L.P.d., Fortunato R.H., Giulietti A.M. (2003). Phylogeny of the Diocleinae (Papilionoideae: Phaseoleae) Based on Morphological Characters. Adv. Legume Syst. Part.

[10] de Queiroz L.P., Pastore J.F.B., Cardoso D., Snak C., Lima A.L.d.C., Gagnon E., Vatanparast M., Holland A.E., Egan A.N. (2015). A Multilocus Phylogenetic Analysis Reveals the Monophyly of a Recircumscribed Papilionoid Legume Tribe Diocleae with Well-Supported Generic Relationships. Mol. Phylogenet Evol..

[11] de Queiroz L.P., Snak C. (2020). Revisiting the Taxonomy of *Dioclea* and Related Genera (Leguminosae, Papilionoideae), with New Generic Circumscriptions. PhytoKeys.

[12] Osterne V.J.S., Santiago M.Q., Pinto-Junior V.R., Cajazeiras J.B., Correia J.L.A., Leitão C.C.F., Carneiro R.F., Pereira-Junior F.N., Vasconcelos M.A., Rocha B.A.M. (2014). Purification, Partial Characterization, and CNBr-Sepharose Immobilization of a Vasorelaxant Glucose/Mannose Lectin from *Canavalia virosa* Seeds. Appl. Biochem. Biotechnol..

[13] Osterne V.J.S., Silva-Filho J.C., Santiago M.Q., Pinto-Junior V.R., Almeida A.C., Barreto A.A.G.C., Wolin I.A.V., Nascimento A.P.M., Amorim R.M.F., Rocha B.A.M. (2017). Structural Characterization of a Lectin from *Canavalia virosa* Seeds with Inflammatory and Cytotoxic Activities. Int. J. Biol. Macromol..

[14] Cavalcante T.T.A., Anderson Matias da Rocha B., Alves Carneiro V., Vassiliepe Sousa Arruda F., Fernandes do Nascimento A.S., Cardoso Sá N., Do Nascimento K.S., Sousa Cavada B., Holanda Teixeira E. (2011). Effect of Lectins from Diocleinae Subtribe against Oral *Streptococci*. Molecules.

[15] Oliveira C.T., Kunz D., Silva C.P., Macedo M.L.R. (2015). Entomotoxic Properties of *Dioclea violacea* Lectin and Its Effects on Digestive Enzymes of *Anagasta kuehniella* (Lepidoptera). J. Insect Physiol..

[16] Vandenborre G., Smagghe G., Van Damme E.J.M. (2011). Plant Lectins as Defense Proteins against Phytophagous Insects. Phytochemistry.

[17] Reyes-Montaño E., Vega Castro N. (2018). Plant Lectins with Insecticidal and Insectistatic Activities. Insecticides-Agriculture and Toxicology.

[18] Macedo M.L.R., Oliveira C.F.R., Oliveira C.T. (2015). Insecticidal Activity of Plant Lectins and Potential Application in Crop Protection. Molecules.

[19] Katoch R., Tripathi A. (2021). Research advances and prospects of legume lectins. J. Biosci..

[20] Mantzoukas S., Korbou G., Magita A., Eliopoulos P.A., Poulas K. (2020). Leguminous Seeds Powder Diet Reduces the Survival and Development of the *Khapra beetle*, *Trogoderma granarium* Everts (Coleoptera: Dermestidae). Biology.

[21] Michiels K., Van Damme E.J., Smagghe G. (2010). Plant-insect interactions: What can we learn from plant lectins?. Arch. Insect Biochem. Physiol.

[22] Fitches E., Wiles D., Douglas A.E., Hinchliffe G., Audsley N., Gatehouse J.A. (2008). The insecticidal activity of recombinant garlic lectins towards aphids. Insect Biochem. Mol. Biol..

[23] Sauvion N., Nardon C., Febvay G., Gatehouse A.M., Rahbé Y. (2004). Binding of the insecticidal lectin Concanavalin A in pea aphid, *Acyrthosiphon pisum* (Harris) and induced effects on the structure of midgut epithelial cells. J. Insect Physiol..

[24] Shukla S., Arora R. (2005). Biological activity of soybean trypsin inhibitor and plant lectins against cotton bollworm/legume pod borer, *Helicoverpa armigera*. Plant Biotechnol..

[25] Sprawka I., Goławska S., Goławski A., Chrzanowski G., Czerniewicz P., Sytykiewicz H. (2013). Entomotoxic action of jackbean lectin (Con A) in bird cherry-oat aphid through the effect on insect enzymes. J. Plant Interact..

[26] Babu R.M., Sajeena A., Seetharaman K., Reddy M.S. (2003). Advances in genetically engineered (transgenic) plants in pest management—An overview. Crop Prot..

[27] Barre A., Van Damme E.J.M., Klonjkowski B., Simplicien M., Sudor J., Benoist H., Rougé P. (2022). Legume Lectins with Different Specificities as Potential Glycan Probes for Pathogenic Enveloped Viruses. Cells.

[28] Fitches E., Edwards M.G., Mee C., Grishin E., Gatehouse A.M.R., Edwards J.P., Gatehouse J.A. (2004). Fusion proteins containing insect-specific toxins as pest control agents: Snowdrop lectin delivers fused insecticidal spider venom toxin to insect haemolymph following oral ingestion. J. Insect Physiol..

[29] Tajne S., Boddupally D., Sadumpati V., Vudem D.R., Khareedu V.R. (2014). Synthetic fusion-protein containing domains of Bt Cry1Ac and *Allium sativum* lectin (ASAL) conferred enhanced insecticidal activity against major lepidopteran pests. J. Biotechnol..

[30] Yang S., Pyati P., Fitches E., Gatehouse J.A. (2014). A recombinant fusion protein containing a spider toxin specific for the insect voltage-gated sodium ion channel shows oral toxicity towards insects of different orders. Insect Biochem. Mol. Biol..

[31] Aksoy S. (2018). Tsetse peritrophic matrix influences for trypanosome transmission. J. Insect Physiol..

[32] Erlandson M.A., Toprak U., Hegedus D.D. (2019). Role of the peritrophic matrix in insect-pathogen interactions. J. Insect physiol..

[33] Konno K., Mitsuhashi W. (2019). The peritrophic membrane as a target of proteins that play important roles in plant defense and microbial attack. J. Insect Physiol..

[34] Sadlova J., Homola M., Myskova J., Jancarova M., Volf P. (2018). Refractoriness of *Sergentomyia schwetzi* to *Leishmania* spp. is mediated by the peritrophic matrix. PLoS Negl. Trop. Dis..

[35] Sede S.M., Tosto D.S., Gottlieb A.M., Poggio L., Fortunato R.H. (2008). %J P. Systematics Genetic Relationships in the *Galactia–Camptosema–Collaea* Complex (Leguminosae) Inferred from AFLP Markers. Plant Syst. Evol..

[36] Burkart A. (1971). El Género *Galactia* (Legum.-Phaseoleae) En Sudamérica Con Especial Referencia a La Argentina y Paises Vecinos. J. Darwiniana.

[37] Moreira Rde A., Ainouz I.L., De Oliveira J.T., Cavada B.S. (1991). Plant Lectins, Chemical and Biological Aspects. Mem. Inst. Oswaldo Cruz.

[38] Rozwarski D.A., Swami B.M., Brewer C.F., Sacchettini J.C. (1998). Crystal Structure of the Lectin from *Dioclea grandiflora* Complexed with Core Trimannoside of Asparagine-Linked Carbohydrates. J. Biol. Chem..

[39] Sanz-Aparicio J., Hermoso J., Grangeiro T.B., Calvete J.J., Cavada B.S. (1997). The Crystal Structure of *Canavalia brasiliensis* Lectin Suggests a Correlation between Its Quaternary Conformation and Its Distinct Biological Properties from Concanavalin A. FEBS Lett..

[40] Wah D.A., Romero A., Gallego del Sol F., Cavada B.S., Ramos M.V., Grangeiro T.B., Sampaio A.H., Calvete J.J. (2001). Crystal Structure of Native and Cd/Cd-Substituted *Dioclea guianensis* Seed Lectin. A Novel Manganese-Binding Site and Structural Basis of Dimer-Tetramer Association. J. Mol. Biol..

[41] Melgarejo L.M., Vega N., Pérez G. (2005). Isolation and characterization of novel lectins from *Canavalia ensiformis* DC and *Dioclea grandiflora* Mart. Ex Benth. seeds. Braz. J. Plant Physiol..

[42] Moreira R.D.A., Cordeiro E.D.F., Ramos V., Grangeiro T.B., Martins J.L., De Oliveira T.A., Cavada B.S., De Ciências C., Federal U. (1996). Isolation and partial characterization of a lectin from seeds of Dioclea violacea. Rev. Bras. Fisiol. Veg..

[43] Bezerra M.J., Rodrigues N.V., Pires Ade F., Bezerra G.A., Nobre C.B., Alencar K.L., Soares P.M., do Nascimento K.S., Nagano C.S., Martins J.L. (2013). Crystal Structure of *Dioclea violacea* Lectin and a Comparative Study of Vasorelaxant Properties with *Dioclea rostrata* Lectin. Int. J. Biochem. Cell Biol..

[44] Loris R., Van Walle I., De Greve H., Beeckmans S., Deboeck F., Wyns L., Bouckaert J. (2004). Structural Basis of Oligomannose Recognition by the *Pterocarpus angolensis* seed lectin. J. Mol. Biol..

[45] Cortázar T.M., Wilson I.B.H., Hykollari A., Reyes E.A., Vega N.A. (2018). Differential Recognition of Natural and Remodeled Glycotopes by Three Diocleae Lectins. Glycoconj. J..

[46] Almanza M., Vega N., Pérez G. (2004). Isolating and Characterising a Lectin from *Galactia lindenii* Seeds That Recognises Blood Group H Determinants. Arch. Biochem..

[47] Queiroz C., Mendes Lopes M.L., Fialho E., Valente-Mesquita V.L. (2008). Polyphenol Oxidase: Characteristics and Mechanisms of Browning Control. Food Rev. Int..

[48] Cortázar T.M. (2019). Estudio Del Efecto de Lectinas Vegetales Sobre Los Procesos de Migración y Proliferación Celular En Queratinocitos Epidérmicos. Ph.D. Thesis.

[49] Fukuda N., Yoshimaru A., Hidaka T., Ohta H., Yamamoto K., Yomo H. (1994). Isolation and Characterization of N-Acetylgalactosamine-Specific Lectin from *Galactia tashiroi* Seeds. Biosci. Biotechnol. Biochem..

[50] Le Pendu J., Gérard G., Lambert F., Mollicone R., Oriol R. (1986). A New Anti-H Lectin from the Seeds of *Galactia tenuiflora*. Glycoconj. J..

[51] Smith P.K., Krohn R.I., Hermanson G.T., Mallia A.K., Gartner F.H., Provenzano M.D., Fujimoto E.K., Goeke N.M., Olson B.J., Klenk D.C. (1985). Measurement of Protein Using Bicinchoninic Acid. Anal. Biochem..

[52] Ceccatto V.M., Cavada B.S., Nunes E.P., Nogueira N.A.P., Grangeiro M.B., Moreno F., Teixeira E.H., Sampaio A.H., Alves M.A.O., Ramos M.V. (2002). Purification and Partial Characterization of a Lectin from *Canavalia grandiflora* Benth. Seeds. Protein Pept. Lett..

[53] Wong J.H., Ng T.B. (2005). Isolation and Characterization of a Glucose/Mannose/Rhamnose-Specific Lectin from the Knife Bean *Canavalia gladiata*. Arch. Biochem. Biophys..

[54] Carrington D.M., Auffret A., Hanke D.E. (1985). Polypeptide Ligation Occurs during Post-Translational Modification of Concanavalin A. Nature.

[55] Pinto-Junior V.R., Osterne V.J., Santiago M.Q., Correia J.L., Pereira-Junior F.N., Leal R.B., Pereira M.G., Chicas L.S., Nagano C.S., Rocha B.A. (2017). Structural Studies of a Vasorelaxant Lectin from *Dioclea reflexa* Hook Seeds: Crystal Structure, Molecular Docking and Dynamics. Int. J. Biol. Macromol..

[56] Reis W.F., Silva M.E.S., Gondim A.C.S., Torres R.C.F., Carneiro R.F., Nagano C.S., Sampaio A.H., Teixeira C.S., Gomes L.C.B.F., Sousa B.L. (2024). Glucose-Binding *Dioclea bicolor* Lectin (DBL): Purification, Characterization, Structural Analysis, and Antibacterial Properties. Protein J..

[57] Correia M.T.S., Coelho L.C.B.B. (1995). Purification of a Glucose/Mannose Specific Lectin, Isoform 1, from Seeds of *Cratylia mollis* Mart.(Camaratu Bean). Appl. Biochem. Biotechnol..

[58] Grangeiro T.B., Gatehouse J.A., Pereira M.N., Cavada B.S. (1997). Investigation on the Origin of the Naturally Occurring Fragments of *Cratylia floribunda* Lectin. Braz. J. Plant Physiol..

[59] Cavada B.S., Marinho E.S., Souza E.P., Benevides R.G., Delatorre P., Souza L.A., Nascimento K.S., Sampaio A.H., Moreno F.B., Rustiguel J.K. (2006). Purification, Partial Characterization and Preliminary X-Ray Diffraction Analysis of a Mannose-Specific Lectin from *Cymbosema roseum* Seeds. Acta Crystallogr. Sect. F Struct. Biol. Cryst. Commun..

[60] Sharon N. (2007). Lectins: Carbohydrate-Specific Reagents and Biological Recognition Molecules. J. Biol. Chem..

[61] Sierra A., Pérez G. (1999). Extracción, Purificación y Caracterización de Dos Lectinas En Semillas de *Dioclea sericea*. Rev. Acad. Colomb. Cienc..

[62] Dam T.K., Cavada B.S., Nagano C.S., Rocha B.A.M., Benevides R.G., Nascimento K.S., De Sousa L.A.G., Oscarson S., Brewer C.F. (2011). Fine Specificities of Two Lectins from *Cymbosema roseum* Seeds: A Lectin Specific for High-Mannose Oligosaccharides and a Lectin Specific for Blood Group H Type II Trisaccharide. Glycobiology.

[63] Dam T.K., Cavada B.S., Grangeiro T.B., Santos C.F., De Sousa F.A.M., Oscarson S., Brewer C.F. (1998). Diocleinae Lectins Are a Group of Proteins with Conserved Binding Sites for the Core Trimannoside of Asparagine-Linked Oligosaccharides and Differential Specificities for Complex Carbohydrates. J. Biol. Chem..

[64] Pérez G. (1998). Isolation and Characterization of a Novel Lectin from *Dioclea lehmannii* (Fabaceae) Seeds. Int. J. Biochem. Cell Biol..

[65] Perez G., Perez C., Sousa-Cavada B., Moreira R., Richardson M. (1991). Comparison of the Amino Acid Sequences of the Lectins from Seeds of *Dioclea lehmannii* and *Canavalia maritima*. Phytochemistry.

[66] Moreira R.A., Monteiro A.C.O., Horta A.C.G., Oliveira J.T.A., Cavada B.S. (1997). Isolation and Characterization of *Dioclea altissima* var. megacarpa Seed Lectin. Phytochemistry.

[67] Artimo P., Jonnalagedda M., Arnold K., Baratin D., Csardi G., de Castro E., Duvaud S., Flegel V., Fortier A., Gasteiger E. (2012). ExPASy: SIB Bioinformatics Resource Portal. Nucleic Acids Res..

[68] Delatorre P., Rocha B.A.M., Souza E.P., Oliveira T.M., Bezerra G.A., Moreno F.B.M.B., Freitas B.T., Santi-Gadelha T., Sampaio A.H., Azevedo W.F. (2007). Structure of a Lectin from *Canavalia gladiata* Seeds: New Structural Insights for Old Molecules. BMC Struct. Biol..

[69] André S., Kaltner H., Manning J.C., Murphy P.V., Gabius H.J. (2015). Lectins: Getting Familiar with Translators of the Sugar Code. Molecules.

[70] Loris R. (2002). Principles of Structures of Animal and Plant Lectins. Biochim. Biophys. Acta (BBA)-Gen. Subj..

[71] Loris R., Hamelryck T., Bouckaert J., Wyns L. (1998). Legume Lectin Structure. Biochim. Biophys. Acta (BBA)-Protein Struct. Mol. Enzymol..

[72] Smith C.K., Regan L. (1997). Construction and Design of β-Sheets. Acc. Chem. Res..

[73] Lagarda-Diaz I., Guzman-Partida A., Vazquez-Moreno L. (2017). Legume Lectins: Proteins with Diverse Applications. Int. J. Mol. Sci..

[74] Chandra N., Prabu M., Suguna K., Vijayan M. (2001). Structural Similarity and Functional Diversity in Proteins Containing the Legume Lectin Fold. Protein Eng..

[75] de Oliveira T.M., Delatorre P., da Rocha B.A.M., de Souza E.P., Nascimento K.S., Bezerra G.A., Moura T.R., Benevides R.G., Bezerra E.H.S., Moreno F.B.M.B. (2008). Crystal Structure of *Dioclea rostrata* Lectin: Insights into Understanding the pH-Dependent Dimer-Tetramer Equilibrium and the Structural Basis for Carbohydrate Recognition in Diocleinae Lectins. J. Struct. Biol..

[76] Srinivas V.R., Reddy G.B., Ahmad N., Swaminathan C.P., Mitra N., Surolia A. (2001). Legume Lectin Family, the “Natural Mutants of the Quaternary State”, Provide Insights into the Relationship between Protein Stability and Oligomerization. Biochim. Biophys. Acta.

[77] Nagano C.S., Calvete J.J., Barettino D., Pérez A., Cavada B.S., Sanz L. (2008). Insights into the Structural Basis of the PH-Dependent Dimer–Tetramer Equilibrium through Crystallographic Analysis of Recombinant Diocleinae Lectins. Biochem. J..

[78] El-Baba T.J., Clemmer D.E. (2019). Solution Thermochemistry of Concanavalin A Tetramer Conformers Measured by Variable-Temperature ESI-IMS-MS. Int. J. Mass. Spectrom..

[79] Goldstein I.J., Poretz R.D., Liener I.E., Sharon N., Goldstein I.J. (1986). Isolation, Physicochemical Characterization, and Carbohydrate-Binding Specificity of Lectins. The Lectins.

[80] Ramos M.V., Moreira R.A., Cavada B.S., de Oliveira J.T.A., Rouge P. (1996). Interaction of Lectins from the Sub-Tribe Diocleinae with Specific Ligands. R. Bras. Fisiol. Veg..

[81] Varki A., Cummings R.D., Aebi M., Packer N.H., Seeberger P.H., Esko J.D., Stanley P., Hart G., Darvill A., Kinoshita T. (2015). Symbol Nomenclature for Graphical Representations of Glycans. Glycobiology.

[82] Gupta D., Oscarson S., Raju T.S., Stanley P., Toone E.J., Brewer C.F. (1996). A Comparison of the Fine Saccharide-binding Specificity of *Dioclea grandiflora* Lectin and Concanavalin A. Eur. J. Biochem..

[83] Richardson M., Campos F.D.A.P., Moreira R.A., Ainouz I.L., Begbie R., Watt W.B., Pusztai A. (1984). The Complete Amino Acid Sequence of the Major α Subunit of the Lectin from the Seeds of *Dioclea grandiflora* (Mart). Eur. J. Biochem..

[84] Barroso-Neto I.L., Delatorre P., Teixeira C.S., Correia J.L.A., Cajazeiras J.B., Pereira R.I., Nascimento K.S., Laranjeira E.P.P., Pires A.F., Assreuy A.M. (2016). Structural Analysis of a *Dioclea sclerocarpa* Lectin: Study on the Vasorelaxant Properties of Dioclea Lectins. J. Int. J. Biol..

[85] Naismith J.H., Field R.A. (1996). Structural basis of trimannoside recognition by concanavalin A. J. Biol. Chem..

[86] Hamshou M., Van Damme E.J.M., Caccia S., Cappelle K., Vandenborre G., Ghesquière B., Gevaert K., Smagghe G. (2013). High Entomotoxicity and Mechanism of the Fungal GalNAc/Gal-Specific *Rhizoctonia solani* Lectin in Pest Insects. J. Insect Physiol..

[87] Candy L., Peumans W.J., Menu-Bouaouiche L., Astoul C.H., Van Damme J., Van Damme E.J.M., Erard M., Rougé P. (2001). The Gal/GalNAc-Specific Lectin from the Plant Pathogenic Basidiomycete *Rhizoctonia solani* Is a Member of the Ricin-B Family. Biochem. Biophys. Res. Commun..

[88] Vranken A.M., Van Damme E.J.M., Allen A.K., Peumans W.J. (1987). Purification and Properties of an N-acetylgalactosamine Specific Lectin from the Plant Pathogenic Fungus *Rhizoctonia solani*. FEBS Lett..

[89] Shahidi-Noghabi S., Van Damme E.J.M., Iga M., Smagghe G. (2010). Exposure of Insect Midgut Cells to *Sambucus nigra* L. Agglutinins I and II Causes Cell Death via Caspase-Dependent Apoptosis. J. Insect Physiol..

[90] Shahidi-Noghabi S., Van Damme E.J.M., Smagghe G. (2008). Carbohydrate-Binding Activity of the Type-2 Ribosome-Inactivating Protein SNA-I from Elderberry (*Sambucus nigra*) Is a Determining Factor for Its Insecticidal Activity. Phytochemistry.

[91] Hamshou M., Van Damme E.J.M., Vandenborre G., Ghesquière B., Trooskens G., Gevaert K., Smagghe G. (2012). GalNAc/Gal-Binding *Rhizoctonia solani* Agglutinin Has Antiproliferative Activity in *Drosophila melanogaster* S2 Cells via MAPK and JAK/STAT Signaling. PLoS ONE.

[92] Tamura T., Sadakata N., Oda T., Muramatsu T. (2002). Role of Zinc Ions in Ricin-Induced Apoptosis in U937 Cells. Toxicol. Lett..

[93] Hughes J.N., Lindsay C.D., Griffiths G.D. (1996). Morphology of Ricin and Abrin Exposed Endothelial Cells Is Consistent with Apoptotic Cell Death. Hum. Exp. Toxicol..

[94] Wolin I.A.V., Heinrich I.A., Nascimento A.P.M., Welter P.G., Sosa L.d.V., De Paul A.L., Zanotto-Filho A., Nedel C.B., Lima L.D., Osterne V.J.S. (2021). ConBr Lectin Modulates MAPKs and Akt Pathways and Triggers Autophagic Glioma Cell Death by a Mechanism Dependent upon Caspase-8 Activation. Biochimie.

[95] de Oliveira Silva F., das Neves Santos P., de Melo C.M.L., Teixeira E.H., de Sousa Cavada B., Pereira V.A.R., Porto A.L.F., Cajazeiras J.B., Arruda F.V.S., Almeida A.C. (2011). Immunostimulatory Activity of ConBr: A Focus on Splenocyte Proliferation and Proliferative Cytokine Secretion. Cell Tissue Researcn.

[96] Cui B., Li L., Zeng Q., Lin F., Yin L., Liao L., Huang M., Wang J. (2017). A Novel Lectin from *Artocarpus lingnanensis* Induces Proliferation and Th1/Th2 Cytokine Secretion through CD45 Signaling Pathway in Human T Lymphocytes. J. Nat. Med..

[97] Wang K., Liu C., Hou Y., Zhou H., Wang X., Mai K., He G. (2019). Differential Apoptotic and Mitogenic Effects of Lectins in Zebrafish. Front. Endocrinol..

[98] Valentiner U., Fabian S., Schumacher U., Leathem A.J. (2003). The Influence of Dietary Lectins on the Cell Proliferation of Human Breast Cancer Cell Lines in Vitro. Anticancer. Res..

[99] Maciel E.V.M., Araújo-Filho V.S., Nakazawa M., Gomes Y.M., Coelho L.C.B.B., Correia M.T.S. (2004). Mitogenic Activity of *Cratylia mollis* Lectin on Human Lymphocytes. Biologicals.

[100] Pani G., Colavitti R., Borrello S., GaleottiI T. (2000). Endogenous Oxygen Radicals Modulate Protein Tyrosine Phosphorylation and JNK-1 Activation in Lectin-Stimulated Thymocytes. Biochem. J..

[101] Barral-Netto M., Santos S.B., Barral A., Moreira L.I.M., Santos C.F., Moreira R.A., Oliveira J.T.A., Cavada B.S. (1992). Human Lymphocyte Stimulation by Legume Lectins from the Diocleae Tribe. J. Immunol. Investig..

[102] Zha X.-L., Wang H., Sun W., Zhang H.-Y., Wen J., Huang X.-Z., Lu C., Shen Y.-H. (2021). Characteristics of the Peritrophic Matrix of the Silkworm, *Bombyx mori* and Factors Influencing Its Formation. Insect.

[103] Rodríguez-de la Noval C., Rodríguez-Cabrera L., Izquierdo L., Espinosa L.A., Hernandez D., Ponce M., Moran-Bertot I., Tellez-Rodríguez P., Borras-Hidalgo O., Huang S. (2019). Functional Expression of a Peritrophin A-like SfPER Protein Is Required for Larval Development in *Spodoptera frugiperda* (Lepidoptera: Noctuidae). Sci. Rep..

[104] de Oliveira C.F.R., de Moura M.C., Napoleão T.H., Paiva P.M.G., Coelho L.C.B.B., Macedo M.L.R. (2017). A Chitin-Binding Lectin from *Moringa oleifera* Seeds (WSMoL) Impairs the Digestive Physiology of the Mediterranean Flour Larvae, *Anagasta kuehniella*. Pestic. Biochem. Physiol..

[105] Latimer G.W. (2023). Microchemical Determination of Nitrogen: Micro-Kjeldahl Method. Official Methods of Analysis of AOAC International.

[106] Laemmli U.K. (1970). Cleavage of Structural Proteins during the Assembly of the Head of Bacteriophage T4. Nature.

[107] Perez G. (1984). Isolation and Characterization of a Lectin from the Seeds of *Erythrina edulis*. J. Phytochem..

[108] Bollag D.M., Edelstein S.J. (1994). Chapter 7: Isoelectric Focusing and Two Dimensional Gel Electrophoresis. Protein Methods.

[109] Mouchahoir T., Schiel J.E. (2018). Development of an LC-MS/MS Peptide Mapping Protocol for the NISTmAb. Anal. Bioanal. Chem..

[110] Altschul S.F., Gish W., Miller W., Myers E.W., Lipman D.J. (1990). Basic Local Alignment Search Tool. J. Mol. Biol..

[111] Robert X., Gouet P. (2014). Deciphering Key Features in Protein Structures with the New ENDscript Server. Nucleic Acids Res..

[112] Morris G.M., Huey R., Lindstrom W., Sanner M.F., Belew R.K., Goodsell D.S., Olson A.J. (2009). AutoDock4 and AutoDockTools4: Automated Docking with Selective Receptor Flexibility. J. Comput. Chem..

[113] Trott O., Olson A.J. (2010). AutoDock Vina: Improving the Speed and Accuracy of Docking with a New Scoring Function, Efficient Optimization, and Multithreading. J. Comput. Chem..

[114] Walski T., Van Damme E.J.M., Smagghe G. (2014). Penetration through the Peritrophic Matrix Is a Key to Lectin Toxicity against *Tribolium castaneum*. J. Insect Physiol..

[115] Wu C.-C., Hsu S.-C., Shih H., Lai M.-Z. (2003). Nuclear Factor of Activated T Cells c Is a Target of P38 Mitogen-Activated Protein Kinase in T Cells. Mol. Cell Biol..

[116] Bezerra G.A., Viertlmayr R., Moura T.R., Delatorre P., Rocha B.A.M., do Nascimento K.S., Figueiredo J.G., Bezerra I.G., Teixeira C.S., Simões R.C. (2014). Structural Studies of an Anti-Inflammatory Lectin from *Canavalia boliviana* Seeds in Complex with Dimannosides. PLoS ONE.

[117] De Souza G.A., Oliveira P.S., Trapani S., Santos A.C.O., Rosa J.C., Laure H.J., Faça V.M., Correia M.T., Tavares G.A., Oliva G. (2003). Amino acid sequence and tertiary structure of *Cratylia mollis* seed lectin. Glycobiology.

[118] Gallego Del Sol F., Cavada B., Calvete J. (2007). Crystal structures of *Cratylia floribunda* seed lectin at acidic and basic pHs. Insights into the structural basis of the pH-dependent dimer–tetramer transition. J. Struct. Biol..

[119] Souza Teixeira C., da Silva H.C., de Moura T.R., Pereira-Júnior F.N., Nascimento K.S.D., Nagano C.S., Sampaio A.H., Delatorre P., Rocha B.A.M., Cavada B.S. (2012). Crystal structure of the lectin of *Camptosema pedicellatum*: Implications of a conservative substitution at the hydrophobic subsite. J. Biochem..

