# Supplementary material for: Characterization of a Novel Galactia lindenii Lectin and Its Effects on Lepidopteran Midgut Cells
Source: Int J Mol Sci. 2025 Oct 24;26(21):10359. doi: 10.3390/ijms262110359 (PMC12607393; doi:10.3390/ijms262110359)
Supplement: Supplementary file 1 [file ijms-26-10359-s001.zip › ijms-3810231-supplementary.pdf]

Supplementary Material

Supplemental Tables S1, S2, S3, S4

Zulma Casas-Corredor [1,2*], Edgar Reyes-Montaño [1,*], Nohora Vega-Castro [1], Mónica Quintero [1], Deisy Hidalgo-Roa [1] and José Luis Fernández-Alonso[3]

1 Grupo de Investigación en Proteínas, Departamento de Química, Facultad de Ciencias, Universidad Nacional de Colombia, Bogotá 111321, Colombia; navegac@unal.edu.co (N.V.-C.); mquintero@unal.edu.co (M.Q.); djhidalgor@unal.edu.co (D.H.-R.)

2 Departamento de Nutrición y Bioquímica. Pontificia Universidad Javeriana. Bogotá. Colombia

3 Real Jardín Botánico CSIC, Biodiversity and Conservation Department, Plaza de Murillo 2, 28014 Ma-drid, España; jlfernandeza@rjb.csic.es (J.L.F-A.)

*** Correspondence: zycasasc@unal.edu.co (Z.C.-C.); eareyesm@unal.edu.co (E.R.-M.)**

Characterization of a Novel *Galactia lindenii* Lectin and Its Effects on Lepidoptera Midgut Cells

(2025)

Table S1. Molecular Properties of Type I Lectins from the *Diocleinae* Tribe

Species/lectin	Inhibitor	Hemagglutination Erythrocytes	Native (kDa)	subunits (kDa)	pI	PDB	References
Dioclea. grandiflora	Man, Glc, Fru	Rabbit	100	α:25 - 26; β:13 - 14; γ: 8 -9	8.6 - 9	1DGL	[38] [83]
D. lehmanni	Man, Glc, Fru, L-sorbose, Me-α-D-Man, Me-α-D-Glc, trehalose	Rabbit, A+, O+, B+	ND	α:25.3; β:14; γ: N.D	8.0 – 8.4	NR	[64], [65]
D. sericea	Man, Glc	A+, O+, B+	57,7	α:29.9; β:16.5; γ: 13.4	6.6 – 6.9	ND	[61]
D. rostrata	Man, Glc, Fru	Rabbit, O+ y B+	ND	α:30.9; β:15.8; γ: 11.7	ND	2ZBJ	[79]
D. altissima	Man, Glc, Fru	Rabbit	100	α:26.3; β:14; γ: 9	8.6 – 9.0	7LJG	[66]
D. lasiophylla	Man, Me-α-D-Man, ovalbumin, fetuin.	Rabbit	ND	α:25.569; β:12.998; γ: 12.588	ND	6CJ9	[55]
D. sclerocarpa	Glc; Gal	Rabbit	102	α: 25.606; β:12.832; γ:12.752	ND	4NOT	[84]
D. violaceae	Man, Glc, Fru, maltose	Rabbit	ND	α:29.5; β:15.8; γ: 11.7	ND	2GDF	[42]
D. reflexa hook	Man, Me-α-D-Man	Rabbit, O+		α: 25.562 ±2; β 12.874 ± 2; γ 12.706 ± 2,	ND	TG3	[55]
D. guianensis	Man, Glc	Rabbit	100	α: 30; β:18; γ:12	ND	1H9P	[40]
Canavalia. ensiformis	Man, Me-α-fructopyranose	Rabbit	96	α:25.5; β:14; γ:12.5	7.1	1JBC	[79]
C. virosa	Man, Glc, α- methyl-D-mannoside.	Rabbit	ND	α:25.48; β:12.86; γ:12.63	ND	5F5Q	[12]
C. brasiliensis	Man, Glc 3,6-di-O- (α-D-Mannopyranosyl-D-mannose)	Horse >Dog	88	α:30; β:16; γ:12	ND	1AZD	[39]
C. marítima	Man, trehalose, maltose	Rabbit, O+	100	α:25; β:14; γ:12	ND	2P34	[80]

C. gladiata	Glc	Rabbit, mouse, human	60	α:30	ND	2OVU	[53]
C. bonariensis	Man	Rabbit	ND	α:25.5; β:13; γ: 12.5	ND	5U3E	[7]
C. boliviana	Man	ND	ND	α:25.57; β:12.87; γ: 12.7	ND	4K21	[116]
Cratylia mollis	Glc, Me-α-D-Man	Rabbit > A+, O+, B+		α:30; β:16; γ: 14	8,5-8,6	1MVQ	[117]
C. floribunda	Man	Rabbit, O+	100	α:25; β:14; γ:12	ND	2D3R 2D3P	[118]
Cymbosema. roseum	Man	Rabbit		α:30; β:18; γ: 12		4MYE	[59]
Captosema pedicellatum	Man, Glc, α- methyl-D-mannopyranoside	Rabbit	ND	α:30; β:16; γ: 13	ND	U4X	[119]
Galactia. lindenii	p-Nitrophenyl-β-D-mannopyranoside, Man	A+, O+	100	29; 60	6,5	NR	This work

Abbreviations. kDa: kilodaltons; pI: isoelectric point; H-Type II: antigen (α-L-Fuc(1-2)-β-D-Gal(1-4)-β-D-GlcNAc-O-R); Man: Mannose.

Glc: Glucose; Me: Methyl; Gal: Galactose; Fru: Fructose; NR: not reported in PDB; ND: undetermined.

Table S2. Inhibition Assessment of GLL-I Activity by Sugars

Carbohydrate	Concentration	Inhibition
D-Glucose	0,150 M	+/-
D-Mannose	0,150 M	+
D-Lactose	0,150 M	-
D-Galactose	0,150 M	+/-
L-Fucose	0,150 M	+/-
GalNAc (N-acetylgalactosamine)	0,150 M	-
1-O-methyl-β-galactopyranoside	0,150 M	+
1-O-methyl-β-glucopyranoside	0,150 M	-
β-methyl D-glucose	0,150 M	+
N-acetyl-α-D-glucosamine	0,150 M	+/-
α-methyl O-mannoside	0,150 M	+/-
D-Galacturonic acid	0,150 M	-
p-Nitrophenyl-β-D-glucopyranoside **	18.5mM	+-
p-Nitrophenyl-β-D-glucosamine **	18.5mM	+/-
p-Nitrophenyl-N-acetyl-α-D-glucosamine **	18.5mM	+
p-Nitrophenyl-β-D-mannopyranoside **	18.5mM	+
p-Nitrophenyl-α-D-mannopyranoside **	18.5mM	+
p-Nitrophenyl-β-D-galactopyranoside **	18.5mM	-
p-Nitrophenyl-N-acetyl-β-D- galactosamine **	18.5mM	+
p-Nitrophenyl-N-acetyl-β-D-galactopyranoside **	18.5mM	-

+: Inhibition agglutination; +/- inhibition is not clear; - not inhibition

**** Soluble up to a concentration of 37 mM**

Table S3. Selected peptide sequences for determining the primary structure of GLL-I.

Peptides	E-value (Alignment)	% Identity
RHIGIDVNSIK	8e-07	100
KWNMQNGKV	2e-06	100
KVGTAHIIYNSVDKR	4e-09	100
RLSAVVSYPNADSATVSYDVDLDNVLPEWVRV	2e-20	100
RVGLSASTGLYKE	2e-07	100
KETNTILSWSFTSKL	1e-08	100
KDLILQGDATTGTDGNLELTRV	2e-13	100
RVSSNGSPQGSSVGRA	2e-08	100
KSPDSHPADGIAFFISNIDSSIPSGSTGRL	5e-19	100
RLLGLFPDAN	2e-06	100

Table S4. Interactions between GLL-I and αD-mannose and D-Manα1-3 [D-Manα1-6] D-Manβ1-4 D-GlcNAcβ1-4 D-GlcNAcβ-OH

**αD Manosa**		
GLL-I Residue/Atom	Ligand/ Atom	Distance (Å)
Tyr100 / N	αDMan / O6	2.93
Asp208 / OD2	αDMan /O6	3.17
Leu99 / N	αDMan /O6	3.11
Leu99 / N	αDMan /O5	3.15
Arg228 / N	αDMan /O2	3.09
**DMan1-3 [DMan1-6] DMan1-4 DGlcNAc1-4 DGlcNAc-OH**		
GLL-I Residue/Atom	Ligand/ Atom	Distance (Å)
Asp16/ OD1	DGlcNAc-OH / O6	3.11
Asp16/ OD1	DGlcNAc1-4 / O6	3.21
Arg28/ NH2	DGlcNAc1-4 / O6	2.88
Arg228/ N	DMan1-6 / O2	2.89
Thr226/ OG1	DMan1-4 / O4	3.34
Thr226/ O	DMan1-4 / O4	2.89
Asp14/ ND2	DMan1-6 / O3	2.8
Tyr12/ OH	DMan/ O6	3.15
Leu99/ N	DMan1-6 / O5	3.22
Asp208/ OD1	DMan1-6 / O3	2.84
